# A Trap-Door Mechanism for Zinc Acquisition by *Streptococcus pneumoniae* AdcA

**DOI:** 10.1128/mBio.01958-20

**Published:** 2021-02-02

**Authors:** Zhenyao Luo, Jacqueline R. Morey, Evelyne Deplazes, Alina Motygullina, Aimee Tan, Katherine Ganio, Stephanie L. Neville, Nikolaos Eleftheriadis, Michael Isselstein, Victoria G. Pederick, James C. Paton, Thorben Cordes, Jeffrey R. Harmer, Bostjan Kobe, Christopher A. McDevitt

**Affiliations:** a School of Chemistry and Molecular Biosciences, University of Queensland, Brisbane, Queensland, Australia; b Australian Infectious Diseases Research Centre, University of Queensland, Brisbane, Queensland, Australia; c Institute for Molecular Bioscience, University of Queensland, Brisbane, Queensland, Australia; d Department of Molecular and Biomedical Science, School of Biological Sciences, University of Adelaide, Adelaide, South Australia, Australia; e School of Life Sciences, University of Technology Sydney, Ultimo, New South Wales, Australia; f School of Pharmacy and Biomedical Sciences, Curtin Health Innovation Research Institute, Curtin University, Bentley, Western Australia, Australia; g Centre for Advanced Imaging, The University of Queensland, St. Lucia, Queensland, Australia; h Department of Microbiology and Immunology, The Peter Doherty Institute for Infection and Immunity, University of Melbourne, Melbourne, Victoria, Australia; i Research Centre for Infectious Diseases, Department of Molecular and Biomedical Science, School of Biological Sciences, University of Adelaide, Adelaide, South Australia, Australia; j Molecular Microscopy Research Group, Zernike Institute for Advanced Materials, University of Groningen, Groningen, The Netherlands; k Physical and Synthetic Biology, Faculty of Biology, Ludwig Maximilians-Universität München, Planegg-Martinsried, Germany; Mississippi State University

**Keywords:** ABC transporter, solute-binding protein, *Streptococcus pneumoniae*, zinc

## Abstract

Zinc is an essential element in all domains of life. Nonetheless, how prokaryotes achieve selective acquisition of zinc from the extracellular environment remains poorly understood. Here, we elucidate a novel mechanism for zinc-binding in AdcA, a solute-binding protein of Streptococcus pneumoniae. Crystal structure analyses reveal the two-domain organization of the protein and show that only the N-terminal domain (AdcA_N_) is necessary for zinc import. Zinc binding induces only minor changes in the global protein conformation of AdcA and stabilizes a highly mobile loop within the AdcA_N_ domain. This loop region, which is conserved in zinc-specific solute-binding proteins, facilitates closure of the AdcA_N_ binding site and is crucial for zinc acquisition. Collectively, these findings elucidate the structural and functional basis of selective zinc uptake in prokaryotes.

## Abstract (precis)

Zinc is an essential nutrient for the virulence of bacterial pathogens such as Streptococcus pneumoniae. Many Gram-positive bacteria use a two-domain lipoprotein for zinc acquisition, but how this class of metal-recruiting proteins acquire zinc and interact with the uptake machinery has remained poorly defined.

## INTRODUCTION

The d-block metal ion zinc (Zn^2+^) is essential for all forms of life. In prokaryotes, Zn^2+^ serves as a crucial structural and/or catalytic cofactor in ca. 5 to 6% of the proteome and is involved in critical processes such as carbon metabolism, competence, and the regulation of DNA transcription (1). The essentiality of Zn^2+^ necessitates that pathogenic organisms scavenge this element from the host environment. Despite the relative abundance of Zn^2+^ in vertebrate hosts, its bioavailability is tightly controlled. To overcome host Zn^2+^ restriction, pathogenic bacteria, including Streptococcus pneumoniae (2, 3), Escherichia coli (4), Pseudomonas aeruginosa (5, 6), Staphylococcus aureus (7), and Salmonella enterica serovar Typhimurium (8), employ high-affinity Zn^2+^ uptake pathways. In animal models of infection, Zn^2+^-scavenging pathways primarily belong to the type II ATP-binding cassette (ABC) family of importers (9) but can also include the zinc-iron permease transporters (10), P-type ATPases (11), and zincophore scavenging systems (7). Bacterial ABC permeases are comprised of an extracytoplasmic solute-binding protein (SBP), which recruits ligands from the bulk solvent, and an ABC transporter, which facilitates cellular import (9). How Zn^2+^-specific SBPs achieve metal ion cargo selection from the complex chemical milieu of the host environment has remained unclear.

S. pneumoniae (the pneumococcus) is the leading cause of bacterial pneumonia, which accounts for 15% of all childhood disease mortalities, and has a global economic health burden of more than US$4 billion annually (12, 13). Zinc is essential for virulence and survival and is acquired solely by the ABC transporter, AdcCB, and two Zn^2+^-specific cluster A-I SBPs, AdcA and AdcAII, in S. pneumoniae (3, 14, 15). AdcA and AdcAII have overlapping functional roles with respect to Zn^2+^ recruitment, with either SBP sufficient for *in vitro* Zn^2+^ homeostasis (2, 3). Nevertheless, both SBPs are required for full virulence, indicating that AdcA and AdcAII have complementary roles during infection (3). The presence of two Zn^2+^-specific SBPs is highly atypical, since most prokaryotes encode only one. However, this arrangement allows AdcA and AdcAII to be differentially regulated in response to Zn^2+^ abundance, thereby enhancing pneumococcal survival *in vivo* (3). AdcA and AdcAII, which share 43% sequence identity over 265 amino acids, belong to the cluster A-I subgroup of SBPs and have a conserved structural fold, comprising a two-lobed organization with N- and C-terminal (β/α)_4_ domains, connected by a rigid lobe-linking α-helix (16–18). The metal-binding site is located within the cleft that bisects the protein surface. Although both SBPs share this structural core, AdcA also has a C-terminal domain (residues 322 to 501; here termed AdcA_C_) that is structurally related to ZinT, a periplasmic protein from E. coli and *Salmonella* (19, 20). ZinT has been implicated in aiding Zn^2+^ uptake, but how this is achieved has remained unclear (19, 20). The AdcA_C_ domain is linked to the N-terminal cluster A-I domain of AdcA (residues 1 to 311, henceforth referred to as AdcA_N_) by an 11-amino-acid linking region (3, 14). AdcA_N_ also has an additional structural feature absent from AdcAII, a region enriched with histidine and glutamate residues (residues 120 to 136, referred to as the His-rich loop). His-rich loops are prevalent in Zn^2+^-specific SBPs, although their contribution to Zn^2+^ recruitment also remains poorly defined (21, 22). Thus, AdcA is comprised of various structural elements implicated in Zn^2+^ acquisition, but how they collectively serve in this process is unknown.

To date, high-resolution crystal structures have only been determined for single domain SBPs from Gram-negative organisms, while two-domain SBPs have remained refractory to such approaches (21, 23, 24). Further, although crystal structures have been solved in the presence or absence of a metal ion, the minor changes in tertiary structure coupled with a lack of insight into protein dynamics have limited mechanistic insight into how Zn^2+^-specific SBPs achieve selection for Zn^2+^ ions. Here, we combined high-resolution structural analyses, molecular dynamics (MD) simulations, and electron paramagnetic resonance (EPR) studies to elucidate the molecular basis of Zn^2+^ acquisition in AdcA. We report the first high-resolution structure of a Zn^2+^-bound two-domain SBP, AdcA, and reveal the essential role of the AdcA_N_ domain in pneumococcal Zn^2+^ uptake and the relative contributions of the “accessory” regions (His-rich loop and AdcA_C_ domain) to this process. We then investigate the conformational landscape of the AdcA_N_ domain and show that ligand-binding induces only localized structural rearrangements within this domain. A key structural element within the AdcA_N_ domain is surface loop α2β2, a highly dynamic region that is stabilized by ligand-binding in a process crucial for AdcCB-mediated Zn^2+^ uptake. Collectively, these findings provide new mechanistic insight into how Zn^2+^-specific cluster A-I SBPs bind metal ligands and the structural elements that contribute to bacterial Zn^2+^ import. These findings provide a structural framework for the development of novel inhibitors targeting AdcA against pneumococcal infections.

## RESULTS

### Structure of Zn^2+^-bound AdcA.

**FIG 1:**
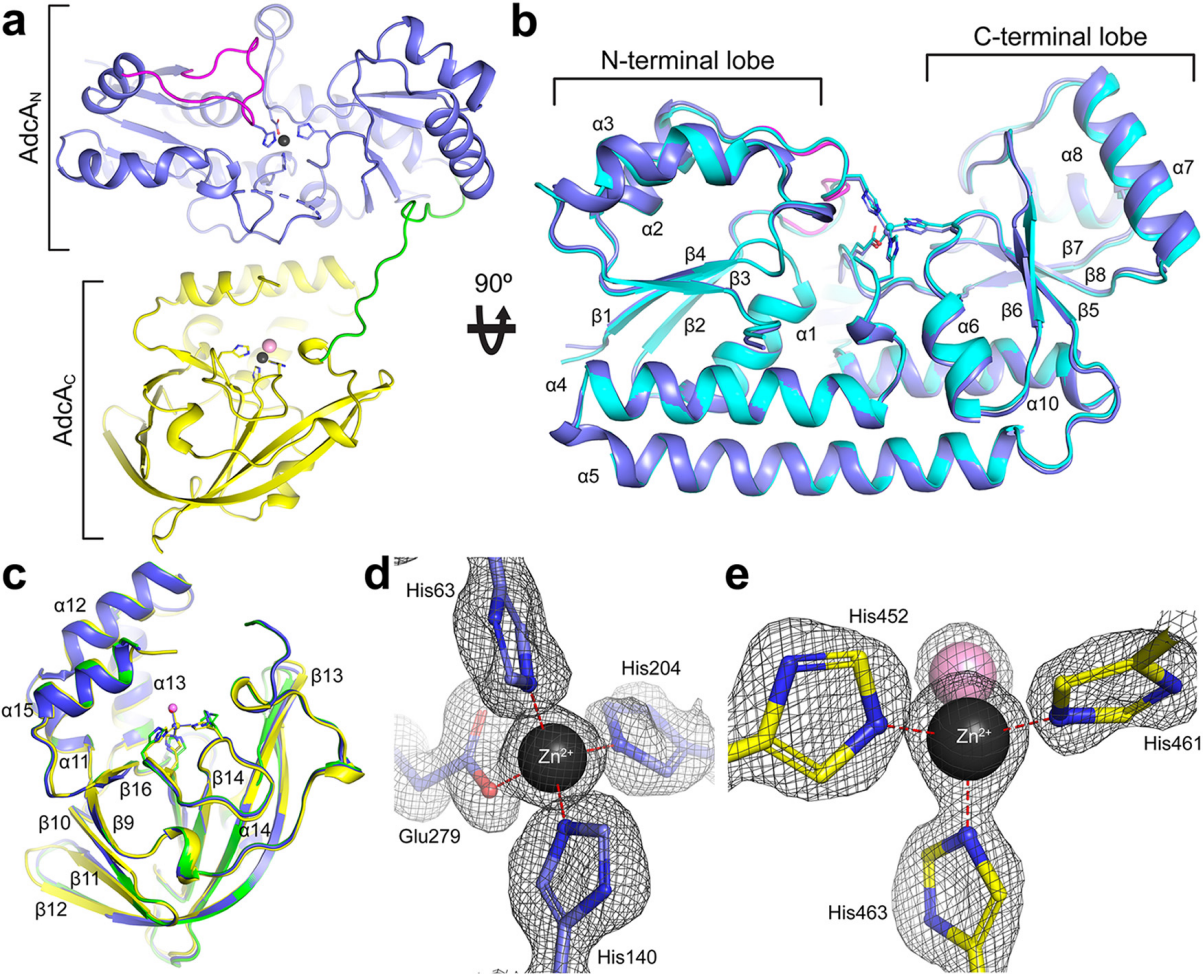
Structure of AdcA. (a) Cartoon representation of the AdcA crystal structure. The AdcA_N_ domain is colored in light blue, and the AdcA_C_ domain is colored in yellow. The bound Zn^2+^ ions are shown as black spheres, with their coordinating residues shown as sticks. The Cl^–^ ion coordinating the Zn^2+^ in the AdcA_C_ domain is shown as a pink sphere. The domain-linking loop is colored in green. The missing residues in the His-rich loop are indicated by a dashed line. The loop α2β2 is colored in magenta. (b) Superposition of the crystal structures of the AdcA_N_ domain from full-length AdcA (light blue) and AdcA_N_ expressed on its own (cyan), viewed from a 90° rotation from the orientation depicted in panel a. The bound Zn^2+^ ions are shown as spheres, with their colors matching the respective structures. The Zn^2+^ coordinating residues are shown as sticks. The coordination bonds are illustrated with dotted lines. (c) Superposition of the crystal structure of the AdcA_C_ domain from full-length AdcA (yellow) with the crystal structures of AdcA_C_ expressed on its own (metal-free, green; Zn^2+^-bound, blue), viewed from the same orientation depicted in panel a. The bound Zn^2+^ are shown as spheres, with their colors matching the respective structures. The Zn^2+^ coordinating residues are shown as sticks. The pink sphere represents the Cl^–^ that is bound to the Zn^2+^ in the AdcA_C_ domain. The coordination bonds are illustrated with dotted lines. (d and e) Close-up views of the metal-binding sites in the AdcA_N_ and AdcA_C_ domains, respectively. The bound Zn^2+^ ions are presented as black spheres, and the coordinating residues are presented as sticks. The coordination bonds are indicated by dashed red lines. The 2F_o_-F_c_ electron density map, contoured at 1.5 σ, is shown in gray mesh presentation.

The high-resolution crystal structure of wild-type, Zn^2+^-bound AdcA was determined at 1.58-Å resolution and revealed a two-domain organization with the AdcA_N_ and AdcA_C_ domains forming discrete globular regions, connected by an 11-amino-acid linker and each containing one Zn^2+^ ion (Fig. 1a to c; see also Fig. S1, Fig. S2, and Table S1 in the supplemental material). The AdcA_N_ domain has a two-lobed organization, with the N- and C-terminal (α/β)_4_ lobes bisected by a cleft in which the metal binds. The binding site in AdcA_N_ is comprised of three Nε2 atoms from His63, His140, and His204 and one Oε2 atom from Glu279. These four residues bind a single Zn^2+^ ion via 4-coordinate geometry, with bond distances of 1.98 to 2.08 Å (Fig. 1d). MD simulations analyzing the stability of the bond distances for the metal-coordinating residues showed that these distances remained stable, averaging 2.07 to 2.15 Å over five independent 750-ns simulations, consistent with Zn^2+^ coordination by proteins (25). Metal binding was restricted to Zn^2+^ ions, with differential scanning fluorimetry (DSF) showing that other divalent first-row transition metal ions did not induce stabilization of the N-terminal domain (Table 1).

**TABLE 1:** Effect of transition metal ions on the melting temperature of AdcA and truncated variants^*a*^

Treatment	AdcA		AdcA_N_		AdcA_C_	
	*T_m_* (°C)	Δ*T_m_* (°C)	*T_m_* (°C)	Δ*T_m_* (°C)	*T_m_* (°C)	Δ*T_m_* (°C)
Metal-free	52.51 ± 0.56		40.74 ± 0.68		54.64 ± 0.50	
Mn^2+^	52.19 ± 0.31	–0.32	40.10 ± 0.02	–0.64	56.31 ± 0.97	+1.67
Fe^2+^	54.55 ± 2.80	+2.04	39.40 ± 0.11	–1.34	55.66 ± 0.16	+1.02
Co^2+^	59.93 ± 1.23	+7.42*	38.93 ± 0.06	–1.81	62.85 ± 1.05	+8.21*
Ni^2+^	60.40 ± 0.65	+7.89*	40.34 ± 0.06	–0.40	63.54 ± 2.13	+8.90*
Cu^2+^	57.57 ± 0.69	+5.06	39.40 ± 0.22	–1.34	57.24 ± 1.12	+2.60
Zn^2+^	66.35 ± 1.78	+13.84*	63.83 ± 0.06	+23.09*	67.80 ± 2.01	+13.16*

a The *T_m_* values represent averages and standard errors of the mean for at least three independent measurements. *, Statistically significant difference compared to metal-free protein *T_m_* (calculated using one-way ANOVA with Tukey’s posttest).

10.1128/mBio.01958-20.2 TABLE S1 Data collection, processing, and refinement statistics for X-ray crystallography. Download Table S1, PDF file, 0.1 MB. Copyright © 2021 Luo et al. 2021 Luo et al. This content is distributed under the terms of the Creative Commons Attribution 4.0 International license.

10.1128/mBio.01958-20.5 FIG S1 Structural analyses of AdcA_N_. Download FIG S1, PDF file, 2.2 MB. Copyright © 2021 Luo et al. 2021 Luo et al. This content is distributed under the terms of the Creative Commons Attribution 4.0 International license.

10.1128/mBio.01958-20.6 FIG S2 Structural analyses of AdcA_C_. Download FIG S2, PDF file, 2.3 MB. Copyright © 2021 Luo et al. 2021 Luo et al. This content is distributed under the terms of the Creative Commons Attribution 4.0 International license.

Electron density was not observed for 14 residues (120 to 133) of the His-rich loop, consistent with the predicted flexibility of this region (26, 27). The two lobes of the AdcA_N_ domain are linked by a rigid α-helix (α5). This fold is highly conserved among cluster A-I SBPs, including S. pneumoniae AdcAII (PDB 3CX3; Cα root mean square deviation [RMSD], 0.91 Å), Salmonella enterica ZnuA (PDB 2XY4; Cα RMSD, 1.78 Å), and S. pneumoniae PsaA (PDB 3ZTT; Cα RMSD, 1.36 Å) (see Fig. S1B in the supplemental material). Intriguingly, the AdcA_N_ domain was observed in a conformation that partially exposed the metal-binding site to bulk solvent, with the relative solvent accessibility of the metal-binding residues (His63 > His140 > His204 ≈ Glu279) remaining the same during the five independent MD simulations. This partial open conformation was also observed in the S. enterica Zn^2+^-bound ZnuA structures (27). However, in other Zn^2+^-bound ZnuA crystal structures, the Zn^2+^-binding sites adopted a fully closed conformation (21, 22, 26). The partial opening of the Zn^2+^-binding site of the AdcA_N_ domain arises from the α7β6 loop having moved away from the binding site due to rigid-body rotational movements of α7 in a plane perpendicular to the helical hinge interlobe helix α5. This conformation was also observed for the truncated AdcA variant that lacked AdcA_C_, AdcA_N_ (residues 27 to 309). The high-resolution crystal structure of Zn^2+^-bound truncated AdcA_N_ (Fig. 1b; see also Fig. S1C and Table S1) did not reveal any conformational differences by comparison with the N-terminal domain of the full-length, wild-type protein.

The AdcA_C_ domain has a lipocalin-like fold of an 8-stranded up-and-down β-barrel (β9-16) and a helical region of four short helices (α11 to α15) (Fig. 1c; see also Fig. S2A). The Zn^2+^-binding site of the AdcA_C_ domain is formed by three Nε2 atoms from His452, His461, and His463 binding a single Zn^2+^ ion with bond distances of 1.97 to 2.11 Å (Fig. 1e). These bond distances remained stable over five independent MD simulations. The metal-binding site of the AdcA_C_ domain showed greater promiscuity than the AdcA_N_ domain, with Co^2+^ and Ni^2+^ inducing protein stabilization, albeit to a lesser extent than Zn^2+^ (Table 1). The overall fold of the AdcA_C_ domain closely resembles the ZinT family of proteins from Gram-negative bacteria, such as E. coli (PDB 1OEK; Cα RMSD, 0.63 Å) and S. enterica (PDB 4AYH; Cα RMSD, 0.58 Å) (see Fig. S2B). The structure of the AdcA_C_ domain was further analyzed compared to a truncated AdcA variant lacking AdcA_N_, AdcA_C_ (residues 326 to 501). The high-resolution crystal structure of isolated Zn^2+^-bound truncated AdcA_C_ did not reveal any conformational differences by comparison with the domain present in the full-length protein (Fig. 1c; see also Fig. S2C and Table S1). The structure of metal-free truncated AdcA_C_ (see Table S1) was also determined, showing that only minor conformational changes are induced by metal binding (Fig. 1c; see also Fig. S2C). These observations are consistent with MD simulations of metal-free and Zn^2+^-bound AdcA; comparisons of Cα RMSD over time showed no significant structural differences between the simulations of the protein in the two states (see Table S2A and Text S1A).

10.1128/mBio.01958-20.1 TEXT S1 (A) Structural differences between MD simulations. (B) Modeling DEER distance distributions. (C) Instrument parameters and data analysis. Download Text S1, PDF file, 0.2 MB. Copyright © 2021 Luo et al. 2021 Luo et al. This content is distributed under the terms of the Creative Commons Attribution 4.0 International license.

10.1128/mBio.01958-20.3 TABLE S2 (A) Cα RMSD values of AdcA, AdcA_N_, and AdcA_C_. (B) Residue pairs forming the interface between AdcA_N_ and AdcA_C_. (C) Hydrogen bond analysis of the lobe-linking α-helix. Download Table S2, PDF file, 0.1 MB. Copyright © 2021 Luo et al. 2021 Luo et al. This content is distributed under the terms of the Creative Commons Attribution 4.0 International license.

In Zn^2+^-bound AdcA, interaction between the domains occurs between α12 and α15 of the AdcA_C_ domain and α7 and the base portion of the His-rich loop (residues 109 to 117) of the AdcA_N_ domain, with a buried surface area of ∼1,200 Å^2^. An extensive array of intramolecular interactions was observed at the interface of the two domains. MD analyses suggest that the most stable portion of the interface arises from interactions between six residues from each domain. The most stable part of the interface involves the salt bridge between Lys368 and Asp213 and H-bonds formed by the side chain of Tyr365 with the backbone and side chain of Asp213 and the backbone of Leu212. Residues Gln492 and Gln496 also form a stable part of the interface, but their interacting residues are dependent upon protein conformation. Gln492 alternates between forming H-bonds with the backbone of Leu115 and the side chain of Asp112. The side chain of Gln496 interacts with both the aromatic ring of Phe137 and the backbone of Leu115. Similarly, Glu120 in the AdcA_N_ domain forms a salt bridge with Lys318, as well as H-bonds with the imidazole ring of His501 in the AdcA_C_ domain, depending on the conformation of the protein. The interactions between these residues define a relatively large and stable buried surface area via a combination of salt bridges, H bonding, and transient interactions. The stability of these interactions and average distances are provided in Table S2B. We then sought to understand how the structural features of AdcA contributed to pneumococcal Zn^2+^ acquisition.

### Zinc acquisition requires the AdcA_N_ domain.

**FIG 2:**
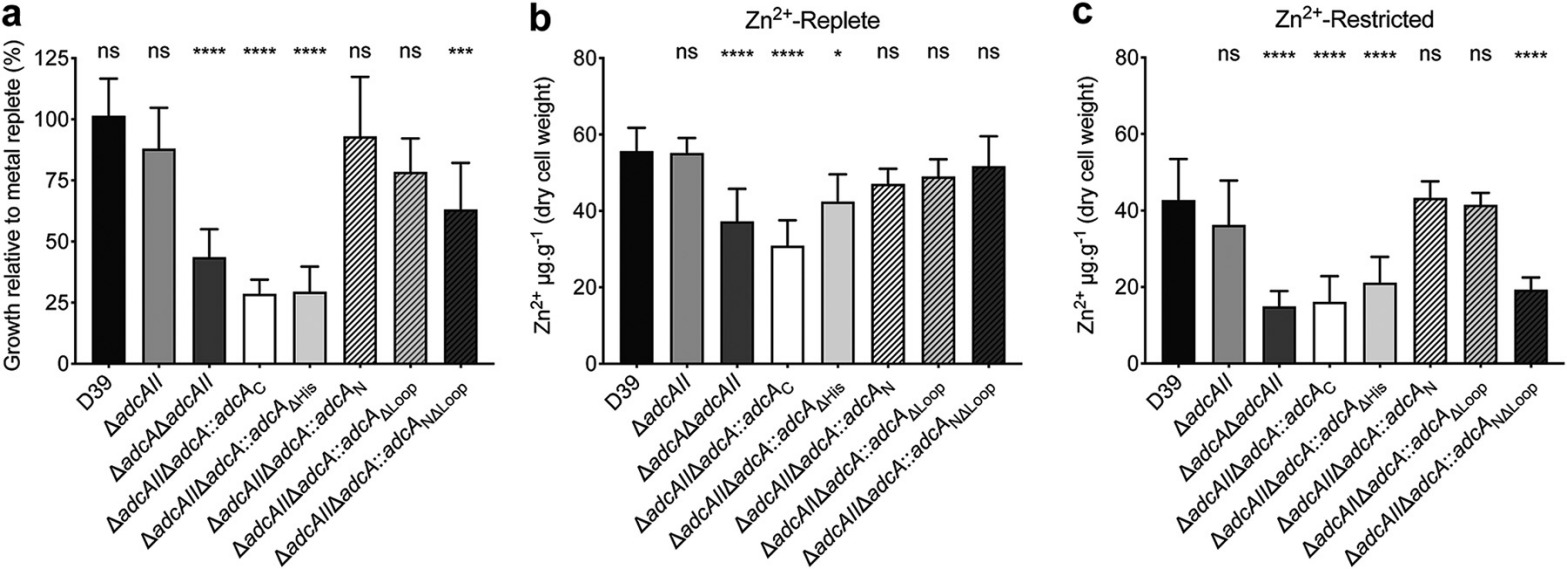
AdcA facilitates growth under Zn^2+^-limiting conditions. (a) *In vitro* growth yields of wild-type S. pneumoniae D39 and the *adc* mutant strains. Bacteria were grown in CDM, with or without treatment with the preferential zinc chelator TPEN (*N*,*N*,*N*′,*N*′-tetrakis(2-pyridinylmethyl)-1,2-ethanediamine). The data are representative mean (± the SEM) optical density at 600 nm (OD_600_) measurements at 300 min from at least three independent biological experiments, normalized to growth in Zn^2+^-replete conditions. Statistical significance of the differences in growth compared to wild-type bacteria was determined by a one-way ANOVA with Tukey posttest. *P* values of <0.005 and <0.0001 are denoted by asterisks (*** or ****, respectively). (b and c) Intracellular Zn^2+^ accumulation of S. pneumoniae D39 and the *adc* mutant strains in CDM in the absence (b) or presence (c) of TPEN supplementation, as determined by ICP-MS. Data correspond to mean (± the SEM) μg Zn^2+^ g^−1^ cell measurements from three independent biological experiments. The statistical significance of the differences in concentrations was determined by using one-way ANOVA with the Tukey posttest. *P* values of <0.05 and <0.0001 are denoted by asterisks (* or ****, respectively). ns, not significant.

The role of the AdcA_N_ domain was investigated using mutant variants of AdcA wherein the N-terminal domain was deleted (*adcA*_C_) or the Zn^2+^-coordinating His residues (His63, His140, and His204) were mutated to Ala (*adcA*_ΔHis_), thereby abolishing its Zn^2+^-binding capacity. The contribution of AdcA_N_ to Zn^2+^ uptake was then assessed in the S. pneumoniae Δ*adcAII* background. The resultant strains (Δ*adcAII* Δ*adcA*::*adcA*_C_ and Δ*adcAII* Δ*adcA*::*adcA*_ΔHis_) showed a significant growth defect in Zn^2+^-restricted conditions (*P* < 0.0001, one-way analysis of variance [ANOVA]) and had a growth profile in Zn^2+^-replete media similar to that of the Δ*adcA* Δ*adcAII* deletion strain, wherein Adc permease function is abrogated (Fig. 2a; see also Fig. S3). Accumulation of Zn^2+^ in the mutant strains was also significantly impaired in both Zn^2+^-replete and Zn^2+^-restricted media (Fig. 2b and c; see also Fig. S4), with no other transition metal ions showing impaired accumulation (Fig. 2b and c; see also Fig. S4). Taken together, these data show that the AdcA_N_ domain is necessary for pneumococcal Zn^2+^ acquisition.

10.1128/mBio.01958-20.7 FIG S3 Phenotypic impact of Zn^2+^-restriction on *S. pneumoniae* growth. Download FIG S3, PDF file, 0.9 MB. Copyright © 2021 Luo et al. 2021 Luo et al. This content is distributed under the terms of the Creative Commons Attribution 4.0 International license.

10.1128/mBio.01958-20.8 FIG S4 Cellular metal accumulation of *S. pneumoniae* and mutant variants. Download FIG S4, PDF file, 0.8 MB. Copyright © 2021 Luo et al. 2021 Luo et al. This content is distributed under the terms of the Creative Commons Attribution 4.0 International license.

### The AdcA_C_ domain and His-rich loop aid in zinc uptake.

We then investigated the contributions of the AdcA_C_ domain and the His-rich loop of the AdcA_N_ domain (residues 120 to 136) in Zn^2+^ uptake. AdcA variants were generated in which (i) the AdcA_C_ domain was truncated (*adcA*_N_), (ii) the His-rich loop was deleted (*adcA*_ΔLoop_), or (iii) both AdcA_C_ and the His-rich loop were deleted (*adcA*_NΔLoop_). In the Δ*adcAII* background, deletion of either accessory region had no significant impact on growth in Zn^2+^-restricted conditions, compared to Zn^2+^-replete conditions (Fig. 2a; see also Fig. S3). However, loss of both accessory regions (*adcA*_NΔLoop_ strain) resulted in a significant growth defect under Zn^2+^-restriction (Fig. 2a; see also Fig. S3). Zinc accumulation in the *adcA*_NΔLoop_ strain was only impaired during growth under Zn^2+^ restriction, while accumulation of other ions was unaffected (Fig. 2b and c; see also Fig. S4). Collectively, our data show that the AdcA_N_ domain is necessary and sufficient for Zn^2+^ acquisition, with the AdcA_C_ domain and the His-rich loop aiding in Zn^2+^ recruitment during growth under Zn^2+^-restricted conditions. Therefore, it logically follows that Zn^2+^ uptake in the Adc permease is regulated by the AdcA_N_ domain of AdcA.

### Zinc binding induces localized conformational changes.

Ligand binding in SBPs is generally associated with protein conformational changes, although the extent of rearrangement varies depending on the intrinsic flexibility of the protein and the identity of ligand(s) (15). Formation of an SBP-ligand complex is a prerequisite for ABC permease-mediated import. We investigated the influence of Zn^2+^-binding on the conformation of the AdcA_N_ domain to determine whether it contributed as a mechanistic determinant in regulating Zn^2+^ uptake. Due to the paucity of mechanistic information on a Zn^2+^-binding mechanism for cluster A-I SBPs, we initially compared AdcA with the cluster A-I manganese (Mn^2+^) ion-recruiting SBP PsaA, for which a ligand-binding mechanism and the relationship of this mechanism to cation import have been reported (18, 28). In PsaA, ligand binding induces the closure of the metal-binding site, facilitated by partial unwinding of the lobe-linking α-helix and the breakage of H-bonds between main-chain N and O atoms in the helix, and relatively large movement of the C-terminal lobe. These movements are reflected by the high root mean square fluctuations (RMSF) in MD simulations (18). Given the similarity in the protein sequence (51% identity across 313 residues) and function, we investigated whether the AdcA_N_ domain used a similar mechanism. Analysis of the residues in the C-terminal lobe of the AdcA_N_ domain, corresponding to those associated with the mechanism in PsaA (residues 225 to 250), revealed that they did not exhibit high RMSF values. Further, examination of the H-bond network of the lobe-linking α-helix (α5, residues 167 to 193) region in Zn^2+^-bound AdcA_N_ showed that all H-bond lengths were ∼3 Å, the ideal range for a helical conformation (29), and the majority (19 of 25 of H-bonds) were present for ≥95% of the simulation time, with a mean distance of ∼2.0 ± 0.2 Å (see Table S2C). Thus, the α-helix appeared stable, with H-bond networks unaffected by metal occupancy status of the protein. These data suggest that the metal-binding mechanism is not associated with a distortion of the lobe-linking α-helix or a large-scale movement of the SBP lobes, indicating a distinct ligand-binding mechanism.

**FIG 3:**
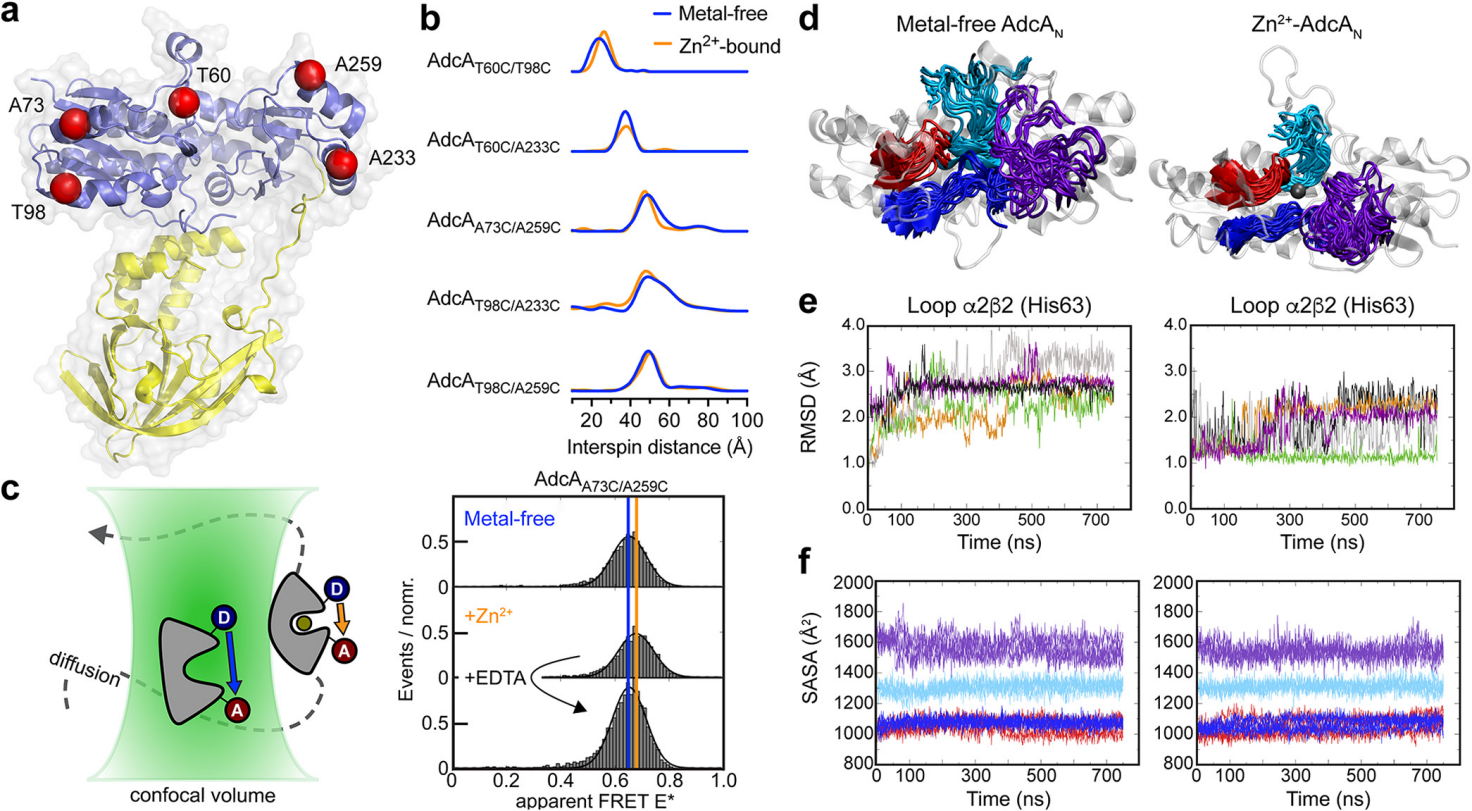
Biophysical and computational analyses of AdcA. (a) Cartoon representation of AdcA, with the five residues mutated to cysteine shown as red spheres. (b) Metal-free (blue) and Zn^2+^-bound (orange) MTSSL-labeled AdcA-Cys variants analyzed by Q-band DEER data, with the distance distributions computed using Tikhonov regularization (see Text S1C). (c) Scheme of the experimental setup for smFRET microscopy of AdcA molecules with donor (D) and acceptor (A). Projections of E* are shown for AdcA_A73C/A259C_ in the metal-free state (blue line; 50 μM EDTA), Zn^2+^-bound state (orange line; 100 μM Zn^2+^) and in the presence of excess EDTA (100 μM Zn^2+^ plus 1 mM EDTA) with fits. (d) AdcA_N_ in the metal-free and Zn^2+^-bound state with snapshots of the four metal-binding residue-containing loops obtained from MD simulations. AdcA_N_ is shown as a cartoon representation (white), with Zn^2+^ as a sphere (black) and the metal-binding residue-containing loops shown in different colors: loop α2β2 (residues 55 to 65; His63) in purple, loop α4β4 (residues 135 to 144; His140) in cyan, loop α6β5 (residues 201 to 207; His204) in red, and loop α10β8 (residues 274 to 281; Glu279) in blue. (e) RMSD analysis of loop α2β2, which contains His63, in the metal-free and Zn^2+^-bound state, with individual trajectories represented by the different colors. RMSD analyses of the other loops are presented in Table 4. (f) Solvent accessible surface area (SASA) over time for the four loops (α2β2, α4β4, α6β5, and α10β8) from the five independent simulations of metal-free and Zn^2+^-bound AdcA, using the loop color scheme in panel d.

The conformational plasticity of the AdcA_N_ domain was then directly examined using double electron-electron resonance (DEER) spectroscopy. Five full-length AdcA variants were generated, by combining pairs of Cys residues in the N-terminal domain of the protein, each of which was analyzed in the metal-free and Zn^2+^-bound states (Fig. 3a). DSF analyses indicated that introduction of the Cys-residues had no impact on Zn^2+^-binding by the AdcA variants (Table 2). DEER determination of the mean NO^•^-NO^•^ distances and widths in Zn^2+^-bound AdcA-Cys variants correlated closely with crystal structure analyses (Fig. 3b and Table 3; see also Text S1B). Nonetheless, the DEER distance distribution widths were slightly greater, suggesting that the protein is slightly more conformationally dynamic in frozen solution. Comparisons of the metal-free and Zn^2+^-bound states of the AdcA-Cys variants revealed only small changes (<4 Å) in the mean distance distributions, as well as small changes in the distance distribution widths (Fig. 3b; see also Fig. S5). We then compared the experimental DEER data with our MD analyses, by deriving an MD structural model of metal-free and Zn^2+^-bound AdcA to describe the DEER data. This was achieved using the complete set of MD trajectories (see Fig. S6A to E) that was then refined to a subset that best fitted the experimental data, resulting in excellent agreement between experiment and model data (Fig. 3b and Table 3; see also Fig. S6F to J). Comparison of the full set of MD simulations computed at room temperature with the subset that optimally describes the frozen-solution DEER data showed that both data sets sampled highly similar conformational landscapes (see Fig. S6K and L). Thus, the experimental DEER data and MD analyses both showed that Zn^2+^-binding in the AdcA_N_ domain induces only minor changes in global protein conformation. To complement these analyses, we investigated full-length AdcA_A73C/A259C_ by smFRET, since the inter-residue distances were compatible with the technique (Fig. 3c). Freely diffusing, fluorophore-labeled AdcA_A73C/A259C_ showed that Zn^2+^-binding induced only a minor change in the apparent FRET efficiency (metal-free E* = 0.65; Zn^2+^-bound E* = 0.68; Fig. 3c) consistent with the DEER and MD observations and similar to our previous smFRET analyses of the SBP PsaA (28). We then investigated the influence of Zn^2+^ on the conformational dynamics of ligand release from AdcA. Here, addition of the divalent-metal chelating compound EDTA immediately removed Zn^2+^ from the AdcA_N_ domain, as shown by return to the apparent FRET efficiencies of the metal-free protein (Fig. 3c). These data indicate that the Zn^2+^-bound conformation of AdcA is able to readily release Zn^2+^ from the high-affinity site in the AdcA_N_ domain to bulk solvent, and thus the lifetime of the AdcA_N_-Zn^2+^ complex is shorter than a few seconds.

**TABLE 2:** Effect of zinc on the melting temperature of AdcA variants

Sample	Mean *T_m_* (°C) ± SEM^*a*^	Δ*T_m_* (°C)^*b*^
Metal-free AdcA_T60C/T98C_	51.26 ± 0.43	
Zn^2+^-AdcA_T60C/T98C_	69.94 ± 0.50	+18.68*
Metal-free AdcA_T60C/A233C_	49.14 ± 1.82	
Zn^2+^-AdcA_T60C/A233C_	68.17 ± 0.50	+19.03*
Metal-free AdcA_A73C/A259C_	48.47 ± 0.47	
Zn^2+^-AdcA_A73C/A259C_	69.60 ± 0.47	+21.13*
Metal-free AdcA_T98C/A233C_	47.37 ± 0.44	
Zn^2+^-AdcA_T98C/A233C_	68.51 ± 1.04	+21.14*
Metal-free AdcA_T98C/A259C_	49.54 ± 0.74	
Zn^2+^-AdcA_T98C/A259C_	68.64 ± 0.50	+20.05*

a The values shown represent averages from at least four independent measurements.

b *, Statistically significant difference compared to the respective metal-free AdcA cysteine variant *T_m_* (one-way ANOVA with Dunnett’s posttest).

**TABLE 3:** Inter-residue distance analyses of AdcA

Protein	C_α_-C_α_ distance (Å) ± the SD		*r*_DD_/σ_DD_ (Å)^*c*^	
	MD^*a*^	MD-DEER^*b*^	DD-crystal	DD-DEER
Metal-free AdcA_T60C/T98C_	22.5 ± 5.8	23.2 ± 6.1		24.9/5.3
Zn^2+^-AdcA_T60C/T98C_	27.7 ± 4.3	21.8 ± 3.8	26.2/5.5	26.5/4.8
Metal-free AdcA_T60C/A233C_	32.7 ± 4.8	33.6 ± 4.5		37.2/3.4
Zn^2+^-AdcA_T60C/A233C_	28.7 ± 3.7	31.5 ± 2.8	34.8/6.4	38.1/6.8
Metal-free AdcA_A73C/A259C_	45.8 ± 3.5	45.4 ± 3.2		52.5/11.1
Zn^2+^-AdcA_A73C/A259C_	44.2 ± 3.1	43.1 ± 2.8	46.6/5.9	49.9/12.1
Metal-free AdcA_T98C/A233C_	46.8 ± 4.5	53.9 ± 5.3		55.4/12.8
Zn^2+^-AdcA_T98C/A233C_	49.9 ± 6.6	50.1 ± 5.4	48.7/6.4	51.8/12.6
Metal-free AdcA_T98C/A259C_	44.7 ± 4.5	52.8 ± 5.4		53.2/17.5
Zn^2+^-AdcA_T98C/A259C_	49.4 ± 6.8	48.2 ± 4.9	49.9/5.9	51.6/15.3

a Calculated mean Cα-Cα distance from the last 250 ns of the five 750-ns MD simulations of AdcA in the metal-bound or metal-free state, as indicated.

b Calculated mean Cα-Cα distances from the set of MD structures that optimally model the DEER data.

c *r*_DD_ is the mean NO^•^-NO^•^ interresidue distance (Å), and σ_DD_ is the width of the DEER distance distribution (DD).

10.1128/mBio.01958-20.9 FIG S5 Q-band DEER traces and distance distributions. Download FIG S5, PDF file, 0.6 MB. Copyright © 2021 Luo et al. 2021 Luo et al. This content is distributed under the terms of the Creative Commons Attribution 4.0 International license.

10.1128/mBio.01958-20.10 FIG S6 DEER and MD structure distance distribution comparisons. Download FIG S6, PDF file, 0.7 MB. Copyright © 2021 Luo et al. 2021 Luo et al. This content is distributed under the terms of the Creative Commons Attribution 4.0 International license.

Collectively, these findings show, for the first time, that Zn^2+^ binding induces only minor changes in the global conformation of AdcA in solution, with the resultant protein-metal complex able to efficiently release bound Zn^2+^ ions. Nevertheless, the ligand-free conformations of AdcA and the AdcA_N_ domain were refractory to crystallization, suggesting that ligand-binding was required to stabilize surface protein dynamics. Thus, we examined the surface dynamics of the AdcA_N_ domain to elucidate their contribution to the Zn^2+^-binding mechanism of the protein.

### Loop α2β2 is a dynamic structural element within AdcA_N_.

The loops containing the metal-coordinating residues are the regions in the AdcA_N_ domain most likely to contain structural elements influenced by Zn^2+^ binding. MD simulations were used to analyze loop mobility, assessed using RMSD and the solvent accessible surface area (SASA) as a function of time in the presence and absence of Zn^2+^ (Fig. 3d to f and Table 4). This revealed that loop α2β2, which contains His63, had the highest mobility and solvent accessibility of the four regions in the metal-free and Zn^2+^-bound states of AdcA (Fig. 3d to f and Table 4). Further, it was the only region to have a significant difference in mobility between the metal-free and Zn^2+^-bound states (Table 4). In contrast, although loop α6β5 (His204) showed some mobility, it did not significantly differ between the metal-free and Zn^2+^-bound states. Loops α10β8 (Glu279) and α4β4 (His140) showed low mobility in either state. These data suggest that His140, His204, and Glu279 provide a largely preformed metal-binding site, with loop α2β2 (His63) acting as a region of dynamic mobility that is stabilized by Zn^2+^ binding.

**TABLE 4:** MD analysis of the AdcA_N_ domain loops containing metal-coordinating residues

AdcA loop	Mean RMSD (Å) ± the SD^*a*^	
	Metal-free AdcA	Zn^2+^-bound AdcA
Loop α2β2 (residues 55–65; His63)	2.7 ± 0.3*	1.9 ± 0.4*
Loop α4β4 (residues 135–144; His140)	1.3 ± 0.3	1.3 ± 0.3
Loop α6β5 (residues 201–207; His204)	2.0 ± 0.2	1.7 ± 0.3
Loop α10β8 (residues 274–281; Glu279)	2.0 ± 0.3	1.6 ± 0.4

a Values shown represent the average Cα RMSD for the AdcA_N_ domain loops containing the metal-binding residues and were calculated by combining the frames from the last 250 ns of each of the five independent 750-ns MD simulations of metal-free and Zn^2+^-bound AdcA, respectively (the data set). The RMSD values were calculated using the starting structure of the simulation as a reference and averaged over the data set. *, Statistically significant difference, based on a 68% confidence interval.

To verify the MD analysis, we investigated the mobility of loop α2β2 in solution, using X-band continuous-wave EPR. Two single AdcA-Cys variants, AdcA_T60C_ (located on loop α2β2) and AdcA_T98C_ (located on helix α3), were examined in the presence and absence of Zn^2+^. We observed that the MTSSL label was more mobile in AdcA_T60C_, by comparison with AdcA_T98C_ (see Fig. S6M), consistent with MD analyses based on RMSD values of these two residues. Upon Zn^2+^ binding, the motility of the MTSSL label on AdcA_T60C_ was reduced to a greater extent than the one on AdcA_T98C_ (see Fig. S6M), consistent with loop α2β2 experiencing a mobility change in response to Zn^2+^-binding. Taken together, these findings indicate that the dynamics of the loop α2β2 are directly influenced by Zn^2+^ binding.

### Role of loop α2β2 in zinc acquisition.

**FIG 4:**
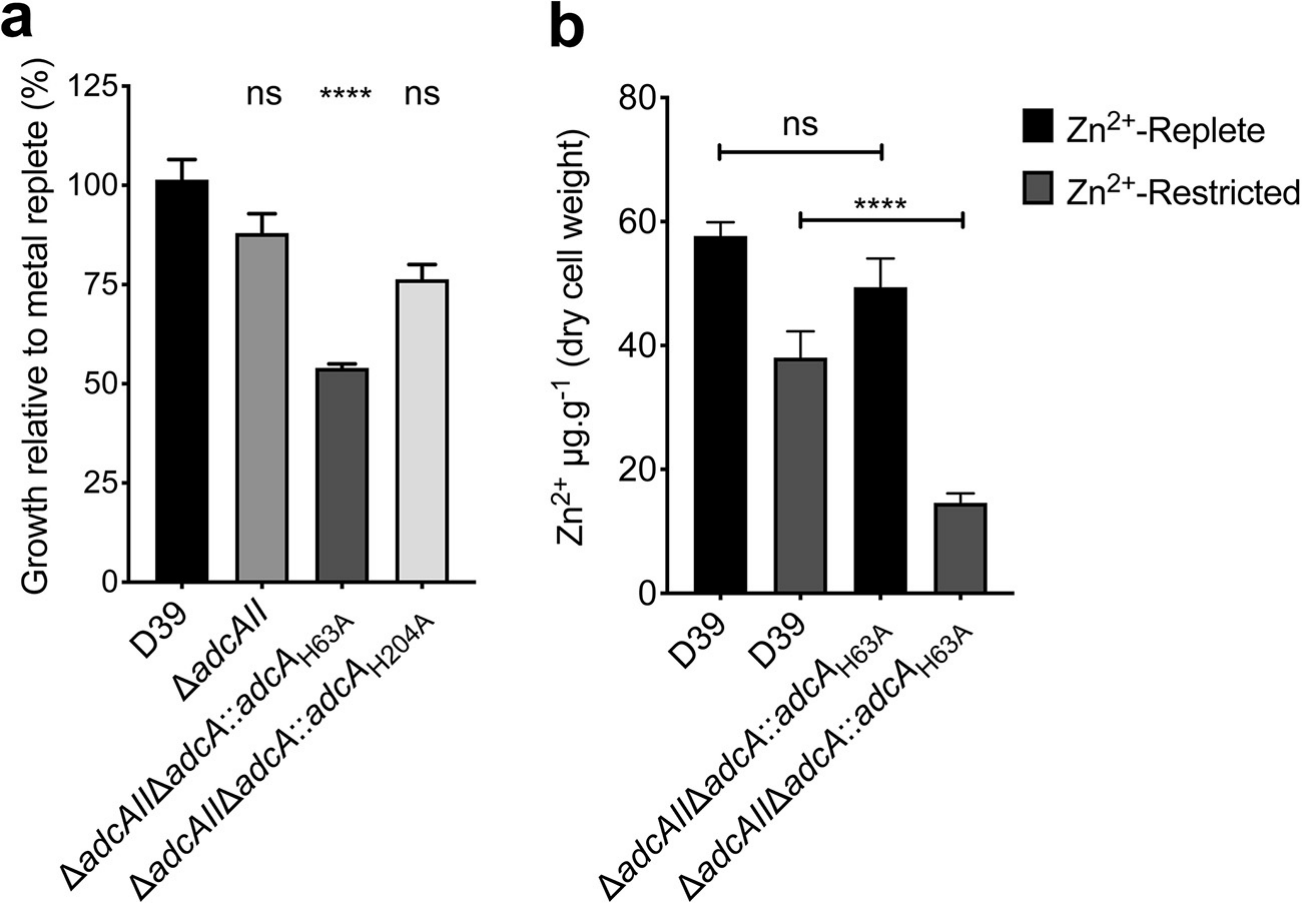
Mutation of His63 impacts Zn^2+^ accumulation. (a) *In vitro* growth measurements of wild-type S. pneumoniae D39 and the Δ*adcAII*, Δ*adcAII* Δ*adcA*::*adcA*_H63A_, and Δ*adcAII* Δ*adcA*::*adcA*_H204A_ mutant strains. Bacteria were grown in CDM, with or without treatment with TPEN, and incubated at 37°C and 5% CO_2_ with growth monitored by optical density (OD_600_) measurements. The data are representative mean (± the SEM) OD_600_ measurements at 300 min from at least three independent biological experiments, normalized to growth in Zn^2+^-replete conditions. The statistical significance of the differences in growth compared to the wild-type strain was determined by a one-way ANOVA, with the Tukey posttest. *P* values of <0.0001 are denoted by asterisks (****). (b) *In vitro* Zn^2+^ accumulation of wild-type S. pneumoniae D39 and Δ*adcAII* Δ*adcA*::*adcA*_H63A_, grown in Zn^2+^-replete or -restricted medium assessed by ICP-MS. The data correspond to the mean (± the SEM) μg metal g^−1^ (dry cell weight) from three measurements in two independent biological experiments. Statistical significance was determined by using one-way ANOVA with the Tukey posttest. *P* values of <0.0001 are denoted by asterisks (****). ns, not significant.

We next investigated the contribution of loop α2β2 in Zn^2+^ acquisition by introducing a H63A mutation to uncouple loop stabilization from Zn^2+^ binding. Mutation of the metal-binding site residue reduced affinity for Zn^2+^, relative to the wild-type protein (3), but was comparable to the impact mediated by mutation of another binding site residue, H204A (Table 5). Introduction of the mutant alleles into S. pneumoniae significantly impacted growth of the Δ*adcAII* Δ*adcA*::*adcA*_H63A_ strain in Zn^2+^-restricted media, relative to the wild-type and Δ*adcAII* strains, but not the Δ*adcAII* Δ*adcA*::*adcA*_H204A_ strain (Fig. 4a). Consistent with the phenotypic growth impact conferred by the mutation of His63, pneumococcal Zn^2+^ accumulation was also reduced in the *adcAII* Δ*adcA*::*adcA*_H63A_ strain, relative to the wild-type strain (Fig. 4b). Thus, while both His63 and His204 contribute to the affinity of AdcA for Zn^2+^ binding, these data indicate that stabilization of loop α2β2 via His63 also contributes to the efficacy of bacterial Zn^2+^ uptake. Taken together, we propose that the dynamic mobility of loop α2β2 contributes to productive interaction of ligand-bound AdcA with the AdcCB transporter to facilitate Zn^2+^ translocation. The molecular details of the SBP-transporter interaction and how it contributes to Zn^2+^ release and import warrant further investigation.

**TABLE 5:** Affinity analyses of truncated AdcA_N_ variants

AdcA_N_ variant	Mean derived *K_d_* (M) ± the SEM^*a*^
H63A	(4.32 ± 0.8) × 10^−7^
H204A	(5.99 ± 1.3) × 10^−7^

a Values represent the means of four replicates.

### Proposed binding mechanism of AdcA.

Collectively, our biochemical, biophysical, and structural analyses reveal how Zn^2+^-specific SBPs selectively acquire Zn^2+^ ions and the contribution of a mobile surface loop (α2β2) in facilitating bacterial metal uptake. In AdcA, the solvent-accessible accessory regions, i.e. the His-rich loop and the AdcA_C_ domain, serve to aid in Zn^2+^ recruitment from bulk solvent and increase its availability to AdcA_N_. Ligand binding in the AdcA_N_ domain occurs at a preformed, relatively static metal-binding site and, although not associated with global changes in protein conformation, stabilizes the surface loop α2β2. In metal-free AdcA, loop α2β2 is highly mobile and increases the solvent accessibility of the metal-binding site. Upon interaction with Zn^2+^, the metal-coordinating residue His63, contained within loop α2β2, initiates closure of the metal-binding site as the loop is pulled onto the binding site, thereby decreasing solvent accessibility. The concomitant reduction in loop α2β2 mobility stabilizes the AdcA_N_ surface. Zinc-bound AdcA is then able to productively interact with the cognate AdcCB transporter and release the bound metal into the translocation pathway. The molecular details of the interaction between AdcA and AdcB and how Zn^2+^ is released to the transporter remain to be elucidated. These structural features, which can be summarized as a limited conformational landscape, a rigid metal-binding site, and a flexible surface loop, appear to be well conserved among Zn^2+^-specific SBPs, suggesting a common mechanistic basis for prokaryotic Zn^2+^ uptake (26, 30). In summary, the metal-binding mechanism in AdcA is akin to a “trap-door,” in which the protein conformational changes can be summarized as a mobile region (loop α2β2, “open trap-door”) that pushes onto a largely static metal-binding site (His140, His204 and Glu279), closing (“shut trap-door”) upon metal binding.

## DISCUSSION

This study defines a structural basis for selective Zn^2+^ acquisition by two-domain lipoproteins in bacterial ABC importers. Our findings show that the AdcA_N_ domain of AdcA is necessary and sufficient for Zn^2+^ import, with ligand binding stabilizing the dynamic loop α2β2. This conformational change contributes to the efficacy of bacterial Zn^2+^ uptake, highlighting the link between ligand-induced conformational changes and the translocation competency of two-domain Zn^2+^-recruiting SBPs. Our mechanism is also consistent with the structural observations with the AdcA_N_ homologs ZnuA from E. coli (26) and SitA from Staphylococcus pseudintermedius (30). Although those studies lacked insight into protein conformational dynamics and Zn^2+^ transport, an analysis of the structural data showed localized conformational rearrangements accompanied by alterations in the flexibility of the loop region containing the residue equivalent to His63. Thus, our findings explain the significance of the Zn^2+^-induced restriction of loop α2β2 mobility. The coupling of ligand-induced localized conformational changes to facile interaction with an ABC transporter is not unprecedented (31). However, it is important to note that the highly localized structural rearrangements in AdcA and other Zn^2+^-specific SBPs are toward the minimal end of the conformational landscape that could facilitate such a process. This is most likely attributable to the structural elements that define the metal-binding sites in AdcA. Nevertheless, while Zn^2+^ binding is intimately linked to protein conformational changes and uptake of the metal ion, it remains to be determined whether these properties influence interaction between AdcA and AdcB.

In the AdcA_N_ domain, the metal-binding site is located beneath the surface of the protein and is largely preformed in the absence of ligand. This permits the bioinorganic chemistry of the site to be tightly defined and likely aids in achieving selectivity for Zn^2+^ over other metal ions. This contrasts starkly with cluster A-I SBPs that interact with a broader range of metal ions, such as S. pneumoniae PsaA and “*Candidatus* Liberibacter asiaticus” ZnuA2 (32). The metal-binding sites in those SBPs offer greater flexibility in metal-ion coordination. However, this plasticity precludes achieving selectivity and is associated with large-scale protein conformational changes upon ligand binding. Thus, the Zn^2+^-binding mechanism used by AdcA is a distinct modality from that used by SBPs that recruit other ions. The metal-binding site in the AdcA_C_ domain is also tightly defined. However, it is highly solvent accessible and is permissive for interaction with Co^2+^ and Ni^2+^ ions, similar to ZinT homologs (33). Despite the interaction of the AdcA_C_ domain with these ions, the AdcA_N_ domain is refractory to their binding, and this prevents their import. These insights are also consistent with the observed role of the accessory regions in enhancing Zn^2+^ uptake during growth in limiting conditions. We note that our observations contradict the recent study of Cao et al. (34), who proposed that streptococcal Zn^2+^ import was more readily facilitated by the AdcA_C_ domain. Our work directly shows that only the AdcA_N_ domain is necessary and essential for Zn^2+^ import. This discrepancy can likely be attributed to their use of a nonnative gene in their complementation analyses. Our conclusions are also consistent with studies from Gram-negative organisms examining the interaction between ZnuA and ZinT homologs (4, 20, 35). However, it is important to note that a direct interaction between ZinT and other proteins *in vivo* has not yet been observed in Gram-negative species. Our structural and computational data reveal that the AdcA_N_ domain and the AdcA_C_ domain form a stable interaction, with the His-rich loop positioned at the interface between the two domains. It is tempting to speculate that the bound Zn^2+^ from the AdcA_C_ domain is transferred to the AdcA_N_ domain Zn^2+^ binding site via the dynamic mobility of the His-rich loop. In this way, the AdcA_C_ domain may enable AdcA to sample Zn^2+^ pools that are spatially inaccessible to the AdcA_N_ domain and thereby increase the efficiency of its acquisition during severe Zn^2+^ restriction. Collectively, these structural and functional analyses provide crucial insights into how accessory regions and proteins aid metal ion receptors to achieve bacterial Zn^2+^ homeostasis.

In conclusion, our work presents a novel “trap-door” mechanism for Zn^2+^ binding in cluster A-I SBPs. Our findings underscore how the ligand-binding sites of Zn^2+^-specific SBPs combine metal specificity with protein-metal interactions. In AdcA, ligand-induced stabilization of highly mobile, local structural elements is crucial for the high-affinity acquisition of Zn^2+^, a poorly abundant metal ion in host tissues that is essential for pneumococcal colonization and disease. The requirement of S. pneumoniae and other pathogens for Zn^2+^ during infection illustrates the therapeutic potential in developing antimicrobials to target crucial, conserved structural elements, such as loop α2β2, and thereby specifically block bacterial Zn^2+^-uptake pathways *in vivo*.

## MATERIALS AND METHODS

### Bacterial strains, culturing, and growth experiments.

Primers used to generate mutant strains, and plasmids for recombinant protein expression are described in Table S3. Bacterial growth experiments were performed with two technical replicates in at least three independent biological experiments, as described previously (18, 36). Growth curves are presented in Fig. S3. Whole-cell metal accumulation was determined by inductively coupled plasma-mass spectrometry (ICP-MS), using an Agilent 8900 ICP-MS/MS using established methods (36, 37).

10.1128/mBio.01958-20.4 TABLE S3 (A) Oligonucleotide primers used in this study. (B) Strains used in this study. (C) Plasmids used in this study. Download Table S3, PDF file, 0.1 MB. Copyright © 2021 Luo et al. 2021 Luo et al. This content is distributed under the terms of the Creative Commons Attribution 4.0 International license.

### Expression, purification, and DSF analysis of AdcA.

Mutant variants of recombinant AdcA were generated by site-directed mutagenesis, using primers listed in Table S3A. Wild-type and mutant AdcA variants were expressed in E. coli LEMO21(DE3) and purified essentially as described previously (3). Protein samples were analyzed for metal content by heating 5 μM protein at 370 K for 15 min in 3.5% HNO_3_, and the metal-ion content was measured by ICP-MS. Differential scanning fluorimetry (DSF) experiments were performed in technical triplicate in at least four independent experiments to determine the mean melting transition (*T_m_*) (36). Fluorescence data (excitation at 470 nm; emission at 570 nm) and the inflection point of the *T_m_* ± (SEM) were calculated using GraphPad Prism (v7.0d). Affinity determination of the mutant AdcA_N_ variants was performed essentially as described previously (38).

### Protein crystallization, crystal structure determination, and structural analyses.

Protein crystals of Zn^2+^-bound AdcA were obtained in 10% (wt/vol) polyethylene glycol (PEG) 20000, 18% (vol/vol) PEG monomethyl ether (MME) 550, 0.03 M CaCl_2_, 0.03 M MgCl_2_, and 0.1 M MES/imidazole (pH 6.5) at 291 K, with a protein concentration of 10 mg ml^−1^ and ZnCl_2_ at a 1:10 protein/Zn^2+^ molar ratio, using the hanging-drop vapor diffusion method. The AdcA_N_ domain fragment was crystallized as described previously (39), and the AdcA_C_ domain fragment was crystallized in 0.1 M sodium acetate (pH 4.5) and 30% (wt/vol) PEG MME 5000 at 293 K. Additional details are provided in Text S1C. Diffraction data collection, processing, and structure refinement statistics can be found in Table S1.

### Electron paramagnetic resonance spectroscopy.

Labeling of the AdcA-Cys variants (10 μM) was performed by incubation with 100 μM *S*-(1-oxyl-2,2,5,5-tetramethyl-2,5-dihydro-1*H*-pyrrol-3-yl)methyl methanesolfonothionate (MTSSL). Free MTSSL was removed by dialysis (10-kDa MWCO) in 1 liter of buffer solution (20 mM morpholinepropanesulfonic acid [pH 7.2], 100 mM NaCl) at 277 K for 24 h. The dialyzed sample was concentrated to 500 μl (10-kDa MWCO) and purified (Superdex 75 Increase 10/300 column). The sample was concentrated to 100 μM (10-kDa MWCO) and flash-frozen (liquid N_2_). X-band CW (continuous wave) EPR spectra in solution were measured on a Bruker Elex E540 spectrometer equipped with a Bruker Super High Sensitivity resonator and a liquid N_2_ temperature control system. Instrument parameters are described in Text S1C. Distance distributions were computed from the DEER time traces with DeerAnalysis (40) using the Tikhonov regularization option and a regularization parameter in the range λ = 100 to 1,000. Tikhonov regularization is a standard mathematical method used to transform the DEER time trace into a model-free distance distribution (i.e., model-free in terms of the shape of the distribution). *In silico* modeling of the spin label rotamer distributions for the metal-free and Zn^2+^-bound protein conformations was computed using MMM 2018.2 (41).

### Molecular dynamics simulations.

The crystal structure of Zn^2+^-bound AdcA was used as the starting structure for all simulations with the set up and parameterization details described in Text S1C. All simulations were carried out using the GROMACS package v5.0.1 (42), in conjunction with the GROMOS 54a7 force field (43) for protein and the simple point charge model for water (44). Analysis was carried out using GROMACS tools. Unless otherwise stated, the five independent simulations for each system were analyzed separately, and only the last 250 ns of each trajectory was used for analysis.

### smFRET microscopy and ALEX.

The smFRET/ALEX technique was adapted from our prior work (45–47). Stochastic labeling of the Cys-AdcA variant AdcA_A73C/A259C_ used the maleimide derivatives of dyes Alexa-555 and Alexa-647, with further details provided in Text S1C. Labeled AdcA_A73C/A259C_ (25 to 100 pM) was studied with smFRET/ALEX at room temperature (50 mM Tris-HCl [pH 7.4], 1 μM EDTA). All experiments were performed using a bespoke confocal microscope assembly as detailed by Husada et al. (45) and as summarized in Text S1C.

### Data availability.

The accession codes for the structures deposited in the Protein Data Bank are as follows: 7JJ9 (Zn^2+^-bound AdcA), 7JJ8 (Zn^2+^-bound AdcA_N_), 7JJA (metal-free AdcA_C_), and 7JJB (Zn^2+^-bound AdcA_C_).
