# Supplementary material for: A Trap-Door Mechanism for Zinc Acquisition by Streptococcus pneumoniae AdcA
Source: mBio. 2021 Feb 2;12(1):e01958-20. doi: 10.1128/mBio.01958-20 (PMC7858048; doi:10.1128/mBio.01958-20)
Supplement: TABLE S1 [file mBio.01958-20-st001.pdf]

Supplementary Table 1. Data collection, processing, and refinement statistics for X-ray crystallography

Data collection				
Diffraction source	Australian Synchrotron MX1 Beamline	Australian Synchrotron MX1 Beamline	Australian Synchrotron MX2 Beamline	Australian Synchrotron MX2 Beamline
Protein crystal	Zn^{2+}-AdcA	Zn^{2+}-$AdcA_N$	$AdcA_C$	Zn^{2+}-$AdcA_C$
Wavelength (Å)	0.954	0.954	0.954	0.954
Resolution range (Å) [a]	19.84-1.58 (1.58-1.61)	19.36-2.03 (2.03-2.10)	19.73-1.01 (1.05-1.01)	19.72-1.1 (1.14-1.10)
Space group	$P\ 1\ 2_1\ 1$	P 1	$P\ 2_1\ 2\ 2_1$	$P\ 2_1\ 2\ 2_1$
Temperature (K)	100	100	100	100
Rotation range per image (°)	0.5	0.5	0.5	0.5
Total rotation range (°)	360	360	360	360
a, b, c (Å)	60.1, 44.4, 92.6	60.4, 68.0, 79.6	44.2, 50.2, 87.8	44.2, 50.1, 87.2
α, β, γ (°)	90.0, 106.8, 90.0	92.3, 104.7, 116.0	90.0, 90.0, 90.0	90.0, 90.0, 90.0
Mosaicity (°)	0.13	0.26	0.09	0.11
Completeness (%)	99.9 (98.7)	98.2 (97.2)	100.0 (100.0)	100.0 (100.0)
R_{merge}	0.096 (0.843)	0.108 (0.804)	0.09 (0.83)	0.03 (0.29)
$\langle I/\sigma(I) \rangle$	15.9 (2.4)	8.6 (1.9)	12.4 (1.8)	13.2 (2.5)
$CC_{1/2}$	0.99 (0.8)	0.99 (0.66)	0.99 (0.64)	0.99 (0.80)
Multiplicity	7.4	3.9	8.0	2.0
No. unique reflections	64356 (275)	68710 (6809)	102911 (5060)	79405 (7855)
R_{work}/R_{free} (%)	16.4/19.9	17.9/22.4	12.8/14.0	14.6/16.7

**No. of non-hydrogen atoms**				
Protein	3680	8352	1508	1873
Ligands	3	4	2	9
Water	669	478	420	360
**Average B-factors (Å^2)**				
Protein	18.9	34.1	12.3	13.3
Ion/ligand	13.2	21.6	16.8	19.1
Waters	28.8	37.1	30.0	27.2
**R.m.s. deviations**				
Bond lengths (Å)	0.009	0.008	0.007	0.007
Bond angles (°)	0.94	0.99	0.94	0.90

[a] Statistics for the highest-resolution shell are shown in parentheses.

$R_{merge} = \sum_{hkl}\sum_{j}|I_{hkl,j} - \langle I_{hkl}\rangle|/(\sum_{hkl}\sum_{j}I_{hkl,j})$

R_{work} / $R_{free} = \sum_{hkl}\left|F_{hkl}^{obs} - F_{hkl}^{calc}\right|/(\sum_{hkl}F_{hkl}^{obs})$; R_{free} was calculated using randomly chosen 10 % fraction of data that was excluded from refinement
