# Supplementary material for: A Trap-Door Mechanism for Zinc Acquisition by Streptococcus pneumoniae AdcA
Source: mBio. 2021 Feb 2;12(1):e01958-20. doi: 10.1128/mBio.01958-20 (PMC7858048; doi:10.1128/mBio.01958-20)
Supplement: FIG S1 [file mBio.01958-20-sf001.pdf]

Supplementary Figures

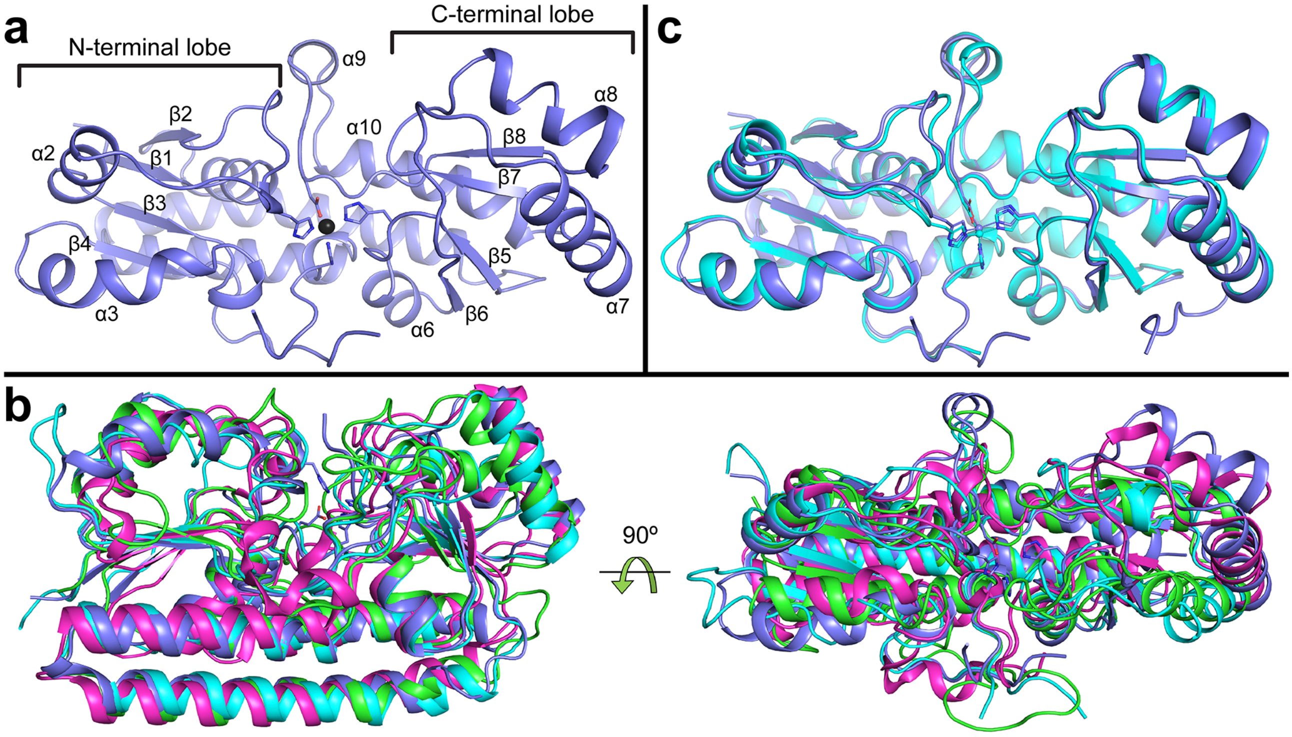

Supplementary Figure 1: Structural analyses of the AdcA$_N$ domain. (**a**) Cartoon representation of the AdcA$_N$ domain. The bound Zn^{2+} ions are shown as black spheres, with their coordinating residues shown as sticks. (**b**) Superposition of the crystal structures of the AdcA$_N$ domain (from full-length AdcA; light blue) with homologous proteins: *S. enterica* ZnuA (PDB accession code: 2XY4, magenta), *S. pneumoniae* PsaA (PDB accession code: 3ZTT, green), and *S. pneumoniae* AdcAII (PDB accession code: 3CX3, cyan). (**c**) Superposition of the crystal structures of the AdcA$_N$ domain from full-length AdcA (light blue) and the AdcA$_N$ domain expressed on its own (cyan). The bound Zn^{2+} are shown as spheres with their colors matching the respective structures. The Zn^{2+} coordinating residues are shown as sticks.
