# Supplementary material for: A Trap-Door Mechanism for Zinc Acquisition by Streptococcus pneumoniae AdcA
Source: mBio. 2021 Feb 2;12(1):e01958-20. doi: 10.1128/mBio.01958-20 (PMC7858048; doi:10.1128/mBio.01958-20)
Supplement: FIG S2 [file mBio.01958-20-sf002.pdf]

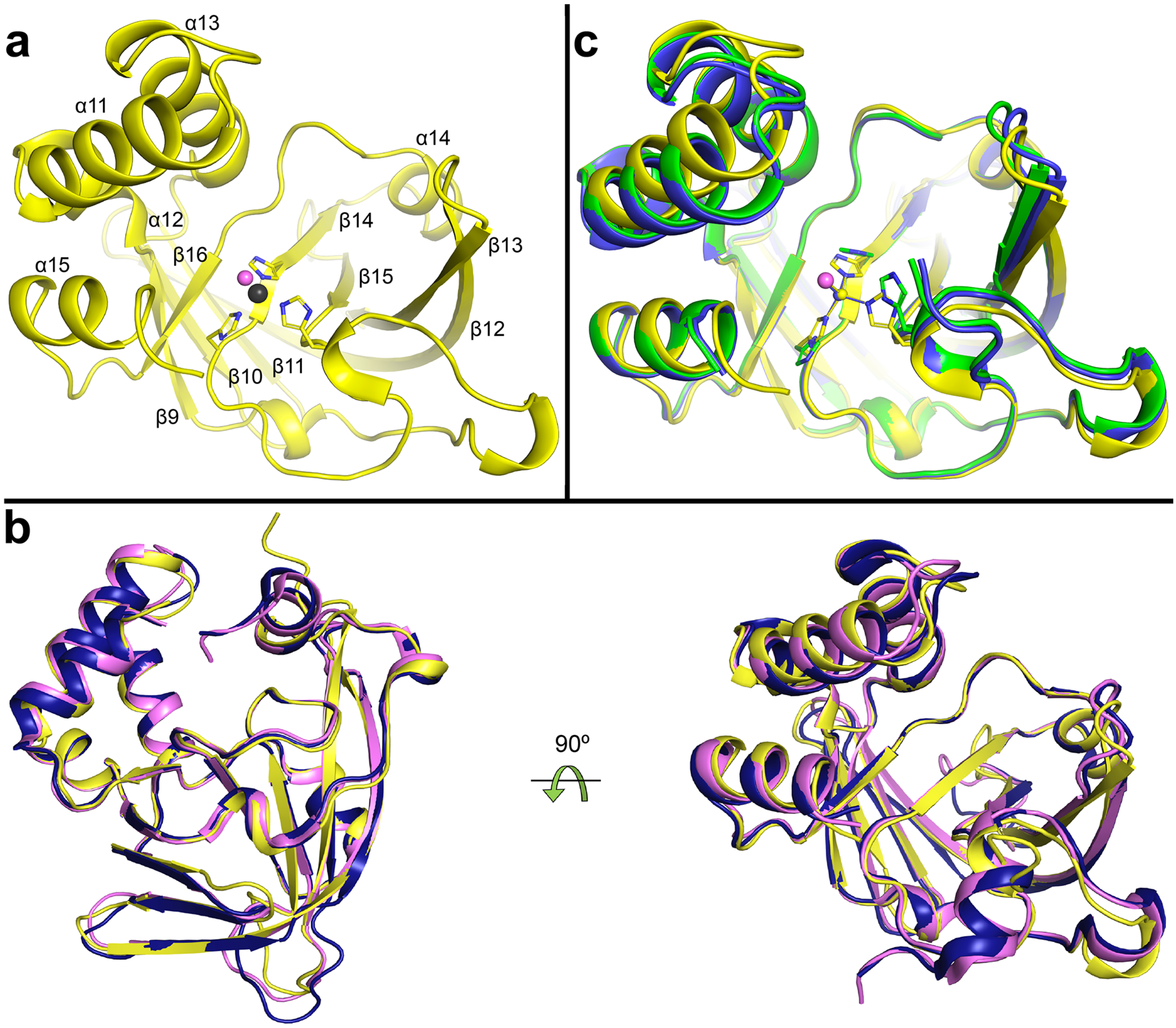

Supplementary Figure 2: Structural analyses of the AdcA$_C$ domain. (**a**) Cartoon representation of the AdcA$_C$ domain. The bound Zn^{2+} ions are shown as black spheres, with their coordinating residues shown as sticks. The coordinating Cl^- ion is shown as pink sphere. The coordination bonds are illustrated with dotted lines. (**b**) Superposition of the crystal structures of the AdcA$_C$ domain (yellow) with homologous proteins, *E. coli* ZinT (PDB accession code: 1OEK, dark blue), *S. enterica* ZinT (PDB accession code: 4AYH, pink). (**c**) Superposition of the crystal structure of the AdcA$_C$ domain from full-length AdcA (yellow) with the crystal structures of the AdcA$_C$ domain expressed on its own (metal-free, green; Zn^{2+}-bound, blue). The bound Zn^{2+} ions are shown as sphere with their colors matching the respective structures. The Zn^{2+} coordinating residues shown as sticks. The coordinating Cl^- ion is shown as a pink sphere.
