# Supplementary material for: A Trap-Door Mechanism for Zinc Acquisition by Streptococcus pneumoniae AdcA
Source: mBio. 2021 Feb 2;12(1):e01958-20. doi: 10.1128/mBio.01958-20 (PMC7858048; doi:10.1128/mBio.01958-20)
Supplement: TEXT S1 [file mBio.01958-20-s0001.pdf]

Supplementary Text 1A. Structural differences between MD simulations.

The Cα RMSD data from **Table S2A** for full-length recombinant AdcA in the metal-bound states shows that the average Cα RMSD from two runs (runs 4,5) differ from the other runs, suggesting that in these runs different protein conformations are sampled. However, further analysis of the $AdcA_N$ domain only (residues 1 to 306) or $AdcA_C$ only (residues 314 to 502) without the loop that connects the $AdcA_N$ and $AdcA_C$ domains (**Table S2A**) reveals that variation of the average Cα RMSD values between the runs of the metal-bound state is reduced, indicating that the difference most likely arises from the linker region. This was further confirmed by clustering analysis showing that differences between the two sets of trajectories lie in the motion of the linking loop and not from conformations sampled by the $AdcA_C$ or $AdcA_N$ domains themselves (data not shown). Based on the Cα RMSD of the $AdcA_C$ or $AdcA_N$ domains only, there is no statistically significant difference between the trajectories from the metal-bound and metal-free simulations.

Supplementary Text 1B. Modelling DEER distance distributions.

The experimental DEER distributions were modelled using a set of structures generated from five independent 750-ns long MD simulations. Each of these conformations was spin labelled *in silico* by attaching the spin label MTSSL and computing rotamers for the residues T60C, T69C, A73C, T98C, A233C, and A259C. The MTSSL rotamers were computed using the molecular modelling software MMM (1) using ambient temperature and the rotamer library R1A. Distance distributions were then computed for the residue pairs of $AdcA_{T60C/T98C}$, $AdcA_{T60C/A233C}$, $AdcA_{A73C/A259C}$, $AdcA_{T98C/A233C}$, $AdcA_{T98C/A259C}$. Each rotamer distance distribution $P(r)$ was normalised to unit area and the five distance distributions for each MD conformation were then arranged into a long column and the set of conformations into a matrix **E**. The experimental distance distributions (each normalised to unit area) were arranged into a long column **y** and a fit to the experimental distance distributions was determined according to $\mathbf{y} = \mathbf{E}\mathbf{c}$ where **c** is a column vector containing the contributions (weightings) of each MD conformation to the modelled distance distribution. Coefficients **c** were determined by

minimisation of the objective function $q = \Sigma(\mathbf{y} - \mathbf{Ec})^2$, subject to the constraint that $\mathbf{c} \geq 0$. This nonnegative linear least-squares problem was solved with using the algorithm described in C. L. Lawson and R. J. Hanson (2) and as implemented in Matlab using the function 'lsqnoneg'. The stability of the solution was checked by cross-validation by removing the structures from each of the five independent MD runs sequentially and examining the conformation space defined by the solution. No significant differences were obtained with respect to the protein conformational space defined by the solution. We additionally examined an algorithm based on an iterative approach described by T. F. Prisner et al. (3) for constructing broad distance distribution from DEER data and the results were again very similar to those obtained from the 'lsqnoneg' algorithm.

Supplementary Text 1C. Instrument parameters and data analysis

Protein crystallization, structure determination, and analyses: Protein crystals of Zn^{2+}-bound AdcA were obtained in 10% w/v polyethylene glycol (PEG) 20000, 18% v/v PEG monomethyl ether (MME) 550, 0.03 M $CaCl_2$, 0.03 M $MgCl_2$, and 0.1 M MES/imidazole pH 6.5 at 291 K, with a protein concentration of 10 mg.mL^{-1} and $ZnCl_2$ at a 1:10 protein to Zn^{2+} molar ratio, using the hanging-drop vapor-diffusion method. The $AdcA_N$ fragment was crystallized as described before (4) and the $AdcA_C$ fragment was crystallized in 0.1 M sodium acetate, pH 4.5, and 30 % (w/v) PEG MME 5000 at 293 K, also using the hanging-drop vapor-diffusion method with a protein concentration of 10 mg.mL^{-1}. Prior to data collection, the crystals were flash-cooled by rapid immersion in liquid nitrogen. The diffraction data were collected on a single crystal at the Australian Synchrotron MX beamlines (5, 6). To determine the structure of AdcA and truncated variants, the diffraction data were indexed and integrated using XDS (7), then scaled and merged in Aimless (8). Initial phases were obtained by molecular replacement using Phenix.Phaser (9), followed by model building in Phenix.AutoBuild (10). The structures were iteratively refined with Phenix.Refine (11) and adjusted manually in Coot (12). Structure validity was assessed using the Molprobity online server (http://molprobity.biochem.duke.edu) (13). Structural analyses (superpositions, metal-ion

coordination and N-/C-terminal domain-crossing angles) were performed in MacPyMOL (Version 1.3 Schrödinger, LLC) and Chimera (14). Data collection, processing, and structure refinement statistics can be found in **Table S1**.

Electron paramagnetic resonance (EPR) spectroscopy: Surface-exposed, non-conserved positions of AdcA were selected for the introduction of cysteine residues for subsequent labelling. Mutant variants were generated by site-directed mutagenesis (Quikchange Lightning Kit, Agilent Technologies) using primers listed in **Table S3A** and produced in *E. coli* LEMO21(DE3) from their respective expression constructs listed in **Table S3C**. Labelling of the recombinant AdcA-Cys variant isoforms (10 μM) was achieved by incubation with 100 μM S-(1-oxyl-2,2,5,5-tetramethyl-2,5-dihydro-1H-pyrrol-3-yl)methyl methanesolfonothionate (MTSSL; Santa Cruz Biotechnology) in a final volume of 1 mL at 277 K for 24 hours under agitation. Free MTSSL was removed by dialysis (10 kDa MWCO SnakeSkin dialysis tubing; Thermo Fisher Scientific) in 1 L of buffer solution (20 mM MOPS pH 7.2, 100 mM NaCl) at 277 K for 24 h. The dialyzed sample was concentrated to 500 μL (10 kDa MWCO Ultra-4 Centrifugal Filter Unit; Amicon) and purified by size-exclusion chromatography (Superdex 75 Increase 10/300 column; GE Healthcare Life Sciences). The purified sample was concentrated to 100 μM (10 kDa MWCO Ultra-4 Centrifugal Filter Unit; Amicon) and the sample loaded into a quartz EPR tube and flash-frozen in liquid N_2 in preparation for EPR measurement.

X-band CW (continuous wave) EPR spectra in solution were measured on a Bruker Elex E540 spectrometer equipped with a Bruker Super High Sensitivity resonator and a N_2 temperature control system (Eurotherm). Measurements were made using a modulation amplitude of 0.2 mT and a modulation frequency of 100 kHz. Four pulse double electron electron resonance (4P DEER) experiments were carried out on a Bruker Elex580 equipped with a Q-band resonator (EN 5107D2, 1.6 mm EPR tubes), a 150 W TWT amplifier (Applied System Engineering Inc., model 187 Ka) and a cryogen free He cryostat (model PT415) held at 55 K. Experiments utilized the detection sub-

sequence $\pi/2 - \tau_1 - \pi - \tau_1 - \tau_2 - \pi - \tau_2$ – echo, with a π pump pulse moved within the first time period ($- \tau_1 - \tau_2 -$). The detection sub-sequence was phase-cycled according to [+x, +x, +x, +; –x, +x, +x, −]. Parameters: $t_{\pi/2}$ = 12-16 ns, t_{π} = 24-32 ns, $t_{\pi,\mathrm{ELDOR}}$ = 16-20 ns and T = 3000 to 5000 ns. The pump pulse was positioned at the maximum of the nitroxide echo signal and the detection pulse frequency lower, $\Delta\nu = (\nu_{det} - \nu_{pump}) = -70$ MHz. Distance distributions were computed from the DEER time traces with the software DeerAnalysis (version 2016) (15) using the Tikhonov regularization option and a regularization parameter in the range λ = 100 - 1000. *In silico* modelling of the MTSSL spin label rotamer distributions for the various metal-free and Zn^{2+} bound protein conformations were computed using MMM 2018.2. (1).

Molecular dynamics simulations: The crystal structure of Zn^{2+}-bound AdcA was used as the starting structure for all simulations. The loop formed by residues 120-133 is missing in the crystal structure and was modelled based on the loop from the structurally related protein PsaA (16). In the crystal structure, the Zn^{2+} ion is coordinated by His63, His140, His204 and Glu279 in the $\mathrm{AdcA_N}$ domain and by His452, His461 and His463 in the $\mathrm{AdcA_C}$ domain. Consistent with the crystal structure, the His residues were modelled with a hydrogen atom on the Nδ1 such that the metal is coordinated by Nε2. For all simulations, AdcA was placed in a rectangular box and solvated with water molecules. Charge was neutralized by adding Na^+ ions and additional Na^+ and Cl^- ions were added to obtain a final ionic strength of 150 mM NaCl. For simulations of Zn^{2+}-free AdcA, the setup was identical except that the two Zn^{2+} ions were removed from the crystal structure. The system was energy-minimized using a steepest descent algorithm. The solvent and protein side-chains were relaxed using a 5 ns simulation in which the protein backbone atoms were position-restrained. This was followed by five independent 750 ns simulations for both the Zn-bound and Zn-free system, respectively.

All simulations were carried out using the GROMACS package version 5.0.1 (17), in conjunction with the GROMOS 54a7 force field (18) for protein and the simple point charge (SPC) model for water (19). Simulations were carried out under periodic boundary conditions with at least

1.5 nm between the protein and the box wall. Non-bonded interactions were described using a twin-range cut-off scheme with a 0.8 nm cut-off for short-range interactions and a 1.4 nm cut-off for long-range interactions. For long-range electrostatic interactions beyond 1.4 nm a reaction field correction was applied using a relative dielectric constant of $\varepsilon = 78.5$, which was developed to be used for simulations with GROMACS and the GROMOS force field. The lengths of covalent bonds were constrained using the SHAKE algorithm, while the geometry of water molecules was constrained using the SETTLE algorithm. Simulations were carried out in the NPT ensemble at T = 298 K and P = 1 bar. The Berendsen thermostat and barostat (20) with coupling constants of 0.1 ps and 0.5 ps were used to maintain the temperature and pressure close to their reference values. For the isotropic pressure coupling, the compressibility was 4.5×10^{-5} bar. Simulations were carried out using a 2-fs time step. Initial velocities were randomly assigned from Maxwellian distributions at 298 K. Configurations were saved every 500 ps for analysis. Analysis was carried out using GROMACS tools. Unless otherwise stated, the five independent simulations for each system were analyzed separately and only the last 250 ns of each trajectory was used for analysis. All images were prepared using VMD (21).

smFRET microscopy and ALEX

The smFRET/ALEX technique was adapted from our prior work (22-24). Stochastic labelling of the Cys-AdcA variant $AdcA_{A73C/A259C}$ used the maleimide derivatives of dyes Alexa555 and Alexa647 (Thermo Fisher Scientific). Purified $AdcA_{A73C/A259C}$, produced as described above, was first pre-treated with 10 mM DTT for 30 min to fully reduce the cysteine residues. The proteins were then immobilized on Ni^{2+}-Sepharose resin (GE Healthcare Life Sciences) and washed with ten column volumes of buffer (50 mM Tris-HCl, pH 7.4, 1 μM EDTA) to remove the DTT. The immobilized proteins were treated with a 5-fold excess of dye and incubated overnight at 277 K. Unbound dye was removed by washing the column with twenty column volumes of buffer, followed by elution of the labelled protein with 400 mM imidazole. The labelled proteins were then purified by size-exclusion

chromatography (Superdex 200, GE Healthcare Life Sciences) achieving a labelling efficiency of >90%.

Labelled $AdcA_{A73C/A259C}$ (25-100 pM) was studied with smFRET/ALEX at room temperature (50 mM Tris-HCl, pH 7.4; 1 μM EDTA). Microscope cover slides were coated with 1 $mg.mL^{-1}$ BSA for 30-60 s to prevent protein absorption to glass (no. 1.5H precision cover slides, VWR). All experiments were performed using a bespoke confocal microscope assembly (detailed in F. Husada et al. (22)). Succinctly, two laser-diodes (Coherent Obis) with emission wavelength of 532 and 637 nm were directly modulated for alternating periods of 50 μs and used for confocal excitation. The laser beams were coupled into a single-mode fiber (PM-S405-XP, Thorlabs) and collimated (MB06, Q-Optics/Linos) before entering a water immersion objective (60×, NA 1.2, UPlanSAPO 60XO, Olympus). The excitation spot was focused 20 μm into the solution. Average laser powers were 30 μW at 532 nm (~30 kW/cm^2) and 15 μW at 637 nm (~15 kW/cm^2). Excitation and emission light were separated by a dichroic beam splitter (zt532/642rpc, AHF Analysentechnik), which was mounted in an inverse microscope body (IX71, Olympus). Emitted light was focused onto a 50 μm pinhole and spectrally separated (640DCXR, AHF Analysentechnik) onto two single-photon avalanche diodes (TAU-SPADs-100, Picoquant) with appropriate spectral filtering (donor channel: HC582/75; acceptor channel: Edge Basic 647LP; AHF Analysentechnik). Photon arrival times in each detection channel were registered by an NI-Card (PXI-6602, National Instruments) and processed using custom software implemented in LabView (National Instruments).

The three relevant photon streams were analyzed (DA, donor-based acceptor emission; DD, donor-based donor emission; AA, acceptor-based acceptor emission) and assignment is based on the excitation period and detection channel. The apparent FRET efficiency is calculated by F(DA)/[F(DA)+F(DD)] and the Stoichiometry S by [F(DD)+F(DA)]/[(F(DD)+F(DA)+F(AA)], where F(·) denotes the summation over all photons within the burst. A dual-color burst search algorithm was used with parameters M = 15, T = 500 μs and L = 25 as described previously (22). In the final histogram only bursts having >150 photons were further analyzed. Data were binned into

FRET histograms (101 x 101 bins) and the selected apparent FRET histograms were analyzed using nonlinear least-square methods as implemented in Origin software; no spectral corrections were done.

References

1. Polyhach Y, Bordignon E, Jeschke G. 2011. Rotamer libraries of spin labelled cysteines for protein studies. Phys Chem Chem Phys 13:2356-66.
2. Lawson CL, Hanson RJ. 1974. Solving Least-Squares Problems. Prentice Hall, Upper Saddle River, New Jersey.
3. Prisner TF, Marko A, Sigurdsson ST. 2015. Conformational dynamics of nucleic acid molecules studied by PELDOR spectroscopy with rigid spin labels. J Magn Reson 252:187-98.
4. Luo Z, Morey JR, McDevitt CA, Kobe B. 2015. Heterogeneous nucleation is required for crystallization of the ZnuA domain of pneumococcal AdcA. Acta Crystallogr F Struct Biol Commun 71:1459-64.
5. Cowieson NP, Aragao D, Clift M, Ericsson DJ, Gee C, Harrop SJ, Mudie N, Panjikar S, Price JR, Riboldi-Tunnicliffe A, Williamson R, Caradoc-Davies T. 2015. MX1: a bending-magnet crystallography beamline serving both chemical and macromolecular crystallography communities at the Australian Synchrotron. J Synchrotron Radiat 22:187-90.
6. Aragao D, Aishima J, Cherukuvada H, Clarken R, Clift M, Cowieson NP, Ericsson DJ, Gee CL, Macedo S, Mudie N, Panjikar S, Price JR, Riboldi-Tunnicliffe A, Rostan R, Williamson R, Caradoc-Davies TT. 2018. MX2: a high-flux undulator microfocus beamline serving both the chemical and macromolecular crystallography communities at the Australian Synchrotron. J Synchrotron Radiat 25:885-891.
7. Kabsch W. 2010. Xds. Acta Crystallogr D Biol Crystallogr 66:125-32.
8. Evans PR, Murshudov GN. 2013. How good are my data and what is the resolution? Acta Cryst D 69:1204-1214.

9. McCoy AJ, Grosse-Kunstleve RW, Adams PD, Winn MD, Storoni LC, Read RJ. 2007. Phaser crystallographic software. J Appl Crystallogr 40:658-674.

10. Terwilliger TC, Grosse-Kunstleve RW, Afonine PV, Moriarty NW, Zwart PH, Hung LW, Read RJ, Adams PD. 2008. Iterative model building, structure refinement and density modification with the PHENIX AutoBuild wizard. Acta Crystallogr D Biol Crystallogr 64:61-9.

11. Afonine PV, Grosse-Kunstleve RW, Echols N, Headd JJ, Moriarty NW, Mustyakimov M, Terwilliger TC, Urzhumtsev A, Zwart PH, Adams PD. 2012. Towards automated crystallographic structure refinement with phenix.refine. Acta Crystallogr D Biol Crystallogr 68:352-67.

12. Emsley P, Lohkamp B, Scott WG, Cowtan K. 2010. Features and development of Coot. Acta Crystallogr D Biol Crystallogr 66:486-501.

13. Chen VB, Arendall WB, 3rd, Headd JJ, Keedy DA, Immormino RM, Kapral GJ, Murray LW, Richardson JS, Richardson DC. 2010. MolProbity: all-atom structure validation for macromolecular crystallography. Acta Cryst D 66:12-21.

14. Pettersen EF, Goddard TD, Huang CC, Couch GS, Greenblatt DM, Meng EC, Ferrin TE. 2004. UCSF Chimera--a visualization system for exploratory research and analysis. J Comput Chem 25:1605-12.

15. Jeschke G, Chechik V, Ionita P, Godt A, Zimmermann H, Banham J, Timmel CR, Hilger D, Jung H. 2006. DeerAnalysis2006—a comprehensive software package for analyzing pulsed ELDOR data. Appl Magn Reson 30:473–498.

16. McDevitt CA, Ogunniyi AD, Valkov E, Lawrence MC, Kobe B, McEwan AG, Paton JC. 2011. A molecular mechanism for bacterial susceptibility to zinc. PLoS Pathog 7:e1002357.

17. Abraham MJ, Murtola T, Schulz R, Páll S, Smith JC, Hess B, Lindahl E. 2015. GROMACS: High performance molecular simulations through multi-level parallelism from laptops to supercomputers. SoftwareX 1-2:19-25.

18. Schmid N, Eichenberger A, Choutko A, Riniker S, Winger M, Mark A, Gunsteren W. 2011. Definition and testing of the GROMOS force-field versions 54A7 and 54B7. Eur Biophys J 40:843-856.

19. Berendsen HJC, Postma JPM, van Gunsteren WF, Hermans J. 1981. Interaction models for water in relation to protein hydration, p 331-342. *In* Pullman B (ed), Intermolecular Forces, vol 14. Springer Netherlands.

20. Berendsen HJC, Postma JPM, van Gunsteren WF, Dinola A, Haak JR. 1984. Molecular dynamics with coupling to an external bath. J Chem Phys 81:3684-3690.

21. Humphrey W, Dalke A, Schulten K. 1996. VMD: visual molecular dynamics. J Mol Graph 14:33-8, 27-8.

22. Husada F, Bountra K, Tassis K, de Boer M, Romano M, Rebuffat S, Beis K, Cordes T. 2018. Conformational dynamics of the ABC transporter McjD seen by single-molecule FRET. EMBO J 37.

23. Jazi AA, Ploetz E, Arizki M, Dhandayuthapani B, Waclawska I, Kramer R, Ziegler C, Cordes T. 2017. Caging and photoactivation in single-molecule Forster resonance energy transfer experiments. Biochemistry 56:2031-2041.

24. Gouridis G, Schuurman-Wolters GK, Ploetz E, Husada F, Vietrov R, de Boer M, Cordes T, Poolman B. 2015. Conformational dynamics in substrate-binding domains influences transport in the ABC importer GlnPQ. Nat Struct Mol Biol 22:57-64.
