# Supplementary material for: A Trap-Door Mechanism for Zinc Acquisition by Streptococcus pneumoniae AdcA
Source: mBio. 2021 Feb 2;12(1):e01958-20. doi: 10.1128/mBio.01958-20 (PMC7858048; doi:10.1128/mBio.01958-20)
Supplement: TABLE S2 [file mBio.01958-20-st002.pdf]

Supplementary Table 2A. C$_{\alpha}$ RMSD values of AdcA, AdcA$_N$ and AdcA$_C$

Run	Metal free AdcA (Å) [a]	Zn^{2+}-bound AdcA (Å) [a]	Metal free AdcA$_N$ (Å) [a]	Zn^{2+}-bound AdcA$_N$ (Å) [a]	Metal free AdcA$_C$ (Å) [a]	Zn^{2+}-bound AdcA$_C$ (Å) [a]
1	5.5 ± 0.2	5.1 ± 0.3	4.7 ± 0.2	3.4 ± 0.3	4.3 ± 0.4	4.4 ± 0.2
2	6.4 ± 0.5	5.4 ± 0.4	3.9 ± 0.3	4.2 ± 0.3	4.7 ± 0.2	4.7 ± 0.2
3	5.8 ± 0.2	4.8 ± 0.3	4.3 ± 0.2	3.4 ± 0.3	3.9 ± 0.2	4.3 ± 0.2
4	6.6 ± 0.2	7.1 ± 0.3	4.3 ± 0.3	4.3 ± 0.2	4.5 ± 0.1	4.4 ± 0.1
5	6.9 ± 0.2	6.6 ± 0.4	5.0 ± 0.2	4.5 ± 0.5	4.3 ± 0.2	4.1 ± 0.2

[a] Values shown represent the average Cα RMSD (± S.D.) calculated by combining the last 250 ns of each of the five independent MD simulations of metal-free and metal-bound state, as indicated. The RMSD for AdcA, AdcA$_N$ and AdcA$_C$ were calculated using the starting structure of the simulation as a reference and averaged over the data set.

Supplementary Table 2B. Residue pairs forming the interface between $AdcA_N$ and $AdcA_C$

Residue pair [a]	Fractional occurrence [b]	Mean distance (Å) [c]
Lys368 (sc) – Asp213 (sc)	0.94	2.4 ± 0.5
Tyr365 (sc) – Leu212 (bb)	0.98	3.0 ± 0.4
Tyr365 (sc) – Asp113 (sc, bb) [d]	0.98	2.0 ± 0.6
Glu120 (sc) – Lys318 (sc)	0.68	2.5 ± 0.6
Glu120 (sc) – His501 (sc, bb) [d]	0.62	2.8 ± 0.7
Gln496 (sc) – Phe137 (sc) [d]	0.67	2.7 ± 0.5
Gln496 (sc) – Leu115 (bb) [d]	0.52	2.6 ± 0.7
Gln492 (sc) – Asp112 (sc)	0.30	2.4 ± 0.7
Gln492 (sc) – Leu115 (bb) [d]	0.55	2.6 ± 0.7

[a.] Residue pairs forming the stable interface between the $AdcA_N$ and $AdcA_C$ domains were calculated using the combined data from the last 250 ns of each of the five independent 750-ns MD simulations of Zn^{2+}-AdcA (the data set). Side chain (sc), backbone (bb).

[b.] Fractional occurrence is the proportion of total frames in the data set in which the minimum distance for the two interacting residues is ≤ 4.0 Å.

[c.] Mean distance represents the mean inter-residue distance (± S.D.) between the residue pairs where inter-residue distance is the minimum distance between any two atoms in the interacting residues, averaged over the frames in the data set in which the interacting occurs (i.e. the frames in which the minimum distance for the two interacting residues is ≤ 4.0 Å and thus contributes to the fractional occurrence).

[d.] Residue pairs in which multiple intra-residue H-bonds (i.e. sc-sc and sc-bb) can be present at the same time.

Supplementary Table 2C: Hydrogen bond analysis of the lobe-linking α-helix

	Metal-free AdcA			Zn^{2+}-bound AdcA		
Hydrogen bond	Fractional occurrence [a]	Mean distance [b]	Mean distance (< 3.0 Å) [c]	Fractional occurrence [a]	Mean distance [b]	Mean distance (< 3.0 Å) [c]
**K167 – K172[d]**	**0.49**	**3.3 ± 1.3**	**2.0 ± 0.2**	**0.77**	**3.6 ± 3.1**	**2.0 ± 0.2**
**E168 – N173**	**0.56**	**3.0 ± 1.1**	**2.0 ± 0.3**	**0.78**	**2.9 ± 1.8**	**1.9 ± 0.2**
T169 – A174	0.73	2.7 ± 1.3	2.0 ± 0.2	1.00	2.0 ± 0.2	2.0 ± 0.2
**F170 – A175**	**0.05**	**4.3 ± 0.5**	**-**	**0.03**	**4.3 ± 0.4**	**-**
E171 – A 175	0.94	2.0 ± 0.5	1.9 ± 0.2	0.98	2.0 ± 0.3	2.0 ± 0.3
K172 – A176	0.93	2.2 ± 0.5	2.0 ± 0.3	0.96	2.0 ± 0.4	2.0 ± 0.3
N173 – Y177	0.99	2.0 ± 0.2	2.0 ± 0.2	0.98	2.2 ± 0.3	2.2 ± 0.3
A174 – I178	1.00	2.0 ± 0.2	2.0 ± 0.2	0.99	2.0 ± 0.2	2.0 ± 0.2
A175 – E179	0.99	2.0 ± 0.2	2.0 ± 0.2	0.99	2.0 ± 0.2	2.0 ± 0.2
A176 – K180	0.96	2.0 ± 0.3	2.0 ± 0.3	0.97	2.1 ± 0.3	2.1 ± 0.3
Y177 – L181	1.00	2.0 ± 0.2	2.0 ± 0.2	0.99	1.9 ± 0.2	1.9 ± 0.2
I178 –Q182	1.00	2.0 ± 0.2	2.0 ± 0.2	0.99	1.9 ± 0.2	1.9 ± 0.2
E179 – S183	0.99	2.0 ± 0.2	2.0 ± 0.2	1.00	2.0 ± 0.2	2.0 ± 0.2
K180 – E190	0.99	2.0 ± 0.2	2.0 ± 0.2	0.99	2.0 ± 0.2	2.0 ± 0.2
L181 – D185	1.00	2.0 ± 0.2	2.0 ± 0.2	1.00	1.9 ± 0.1	1.9 ± 0.1
Q182 – K186	0.64	2.8 ± 1.0	2.0 ± 0.2	0.99	2.0 ± 0.2	2.0 ± 0.2
S183 – A187	1.00	2.0 ± 0.2	2.0 ± 0.2	0.79	2.6 ± 0.8	2.2 ± 0.2
L184 – Y188	0.92	2.2 ± 0.5	2.0 ± 0.3	0.96	2.2 ± 0.3	2.2 ± 0.3
D185 – A189	0.99	2.0 ± 0.2	2.0 ± 0.2	0.99	2.0 ± 0.2	2.0 ± 0.2
K186 – E190	1.00	2.0 ± 0.2	2.0 ± 0.2	1.00	1.9 ± 0.2	1.9 ± 0.2

A187 – G191	0.80	2.5 ± 1.0	2.1 ± 0.3	0.99	1.9 ± 0.2	1.9 ± 0.2
Y188 – L192	0.99	2.0 ± 0.2	2.0 ± 0.2	0.99	2.0 ± 0.2	2.0 ± 0.2
**A189 – S193**	**0.62**	**3.8 ± 2.5**	**2.0 ± 0.3**	**0.58**	**3.1 ± 1.3**	**2.1 ± 0.3**
**E190 – Q194**	**0.46**	**4.0 ± 1.9**	**2.2 ± 0.3**	**0.57**	**3.6 ± 1.9**	**2.0 ± 0.2**

[a] Hydrogen bonds in the inter-lobe linking helix (residues K167 – S193, helix α5) were analysed by combining the frames from the last 250 ns of each of the five independent 750-ns MD simulations of metal-free and metal-bound AdcA, respectively (the data set). Fractional occurrence of H-bonds was calculated as the proportion of total frames in the data set in which the H-bond was ≤ 3.0 Å, based on the distance between the hydrogen bonding atoms.

[b.] Mean distance represents the mean distance between the hydrogen bonding atoms (± S.D.), averaged over all frames in the data set.

[c.] 'Mean distance ≤ 3.0 Å' is the distance between the hydrogen bonding atoms (± S.D.), averaged over frames in which the minimum distance for interaction between the two residues is ≤ 3.0 Å (i.e. averaged over frames where a hydrogen bond is present and thus contributes to the fractional occurrence).

[d.] Residue pairs highlighted in **bold** represent H-bonds with a low mean fractional occurrence (< 0.7) in both the metal-free and metal-bound AdcA MD simulations.
