# Supplementary material for: A Trap-Door Mechanism for Zinc Acquisition by Streptococcus pneumoniae AdcA
Source: mBio. 2021 Feb 2;12(1):e01958-20. doi: 10.1128/mBio.01958-20 (PMC7858048; doi:10.1128/mBio.01958-20)
Supplement: FIG S3 [file mBio.01958-20-sf003.pdf]

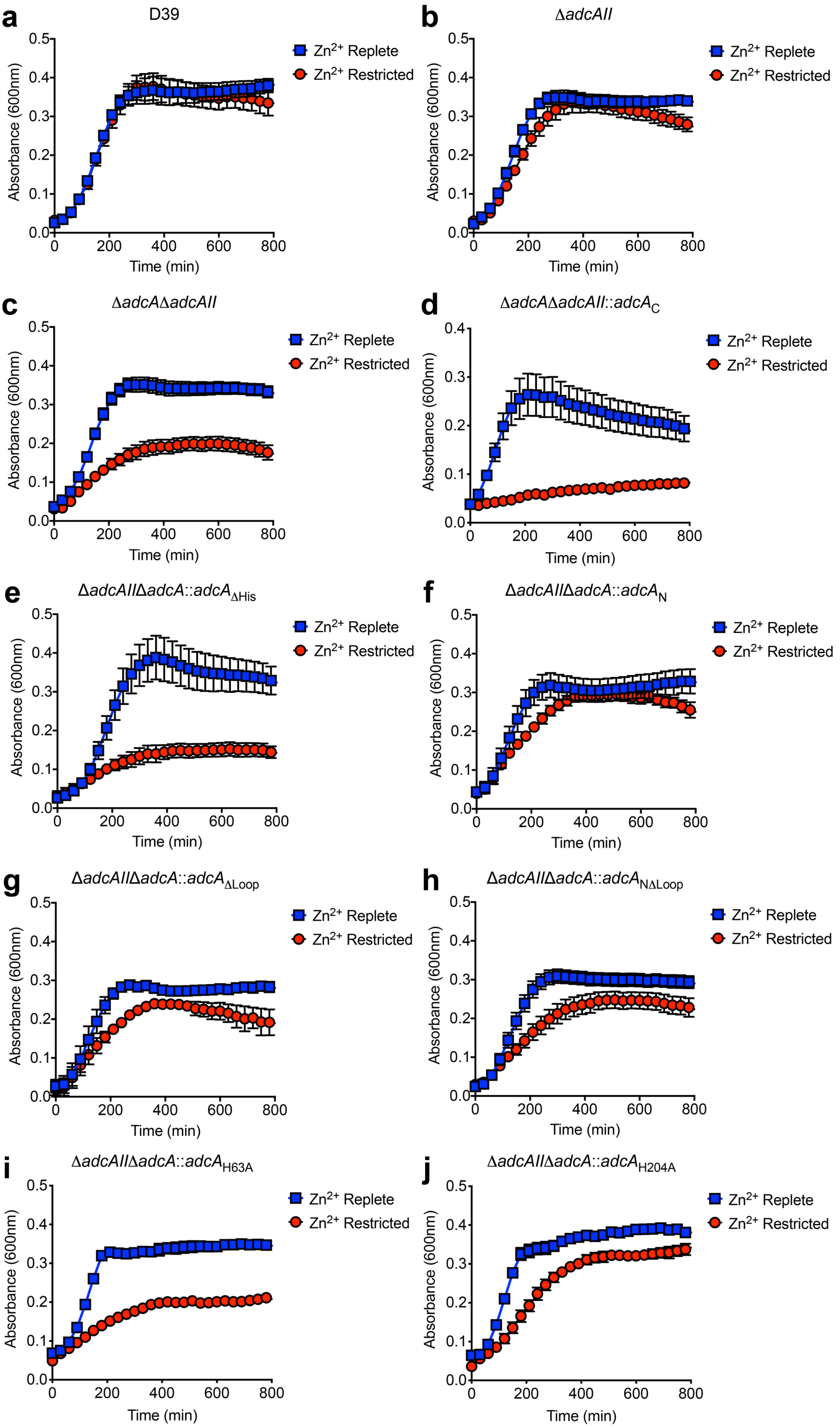
a
D39
b
ΔadcAII
c
ΔadcAΔadcAII
d
ΔadcAΔadcAII::adcA_C
e
ΔadcAIIΔadcA::adcA_ΔHis
f
ΔadcAIIΔadcA::adcA_N
g
ΔadcAIIΔadcA::adcA_ΔLoop
h
ΔadcAIIΔadcA::adcA_NΔLoop
i
ΔadcAIIΔadcA::adcA_H63A
j
ΔadcAIIΔadcA::adcA_H204A
Zn2+ Replete
Zn2+ Restricted
Absorbance (600nm)
Time (min)
0.0
0.1
0.2
0.3
0.4
0.5
0
200
400
600
800

Supplementary Figure 3: Phenotypic impact of Zn^{2+}-restriction on *S. pneumoniae* growth. *S. pneumoniae* D39 (**a**), Δ*adcAII* (**b**), Δ*adcAII*Δ*adcA* (**c**), Δ*adcAII*Δ*adcA*::*adcA*$_{\text{C}}$ (**d**), Δ*adcAII*Δ*adcA*::*adcA*$_{\Delta\text{His}}$ (**e**), Δ*adcAII*Δ*adcA*::*adcA*$_{\text{N}}$ (**f**), Δ*adcAII*Δ*adcA*::*adcA*$_{\Delta\text{Loop}}$ (**g**), Δ*adcAII*Δ*adcA*::*adcA*$_{\text{N}\Delta\text{Loop}}$ (**h**), Δ*adcAII*Δ*adcA*::*adcA*$_{\text{H63A}}$ (**i**), and Δ*adcAII*Δ*adcA*::*adcA*$_{\text{H204A}}$ (**j**) grown in Zn^{2+}-restricted or Zn^{2+}-replete CDM as indicated. The data correspond to mean (± S.E.M.) absorbance 600 nm measurements from at least three independent biological experiments. Error bars, where not visible, are overlapped by the representative symbols.
