# Supplementary material for: A Trap-Door Mechanism for Zinc Acquisition by Streptococcus pneumoniae AdcA
Source: mBio. 2021 Feb 2;12(1):e01958-20. doi: 10.1128/mBio.01958-20 (PMC7858048; doi:10.1128/mBio.01958-20)
Supplement: FIG S4 [file mBio.01958-20-sf004.pdf]

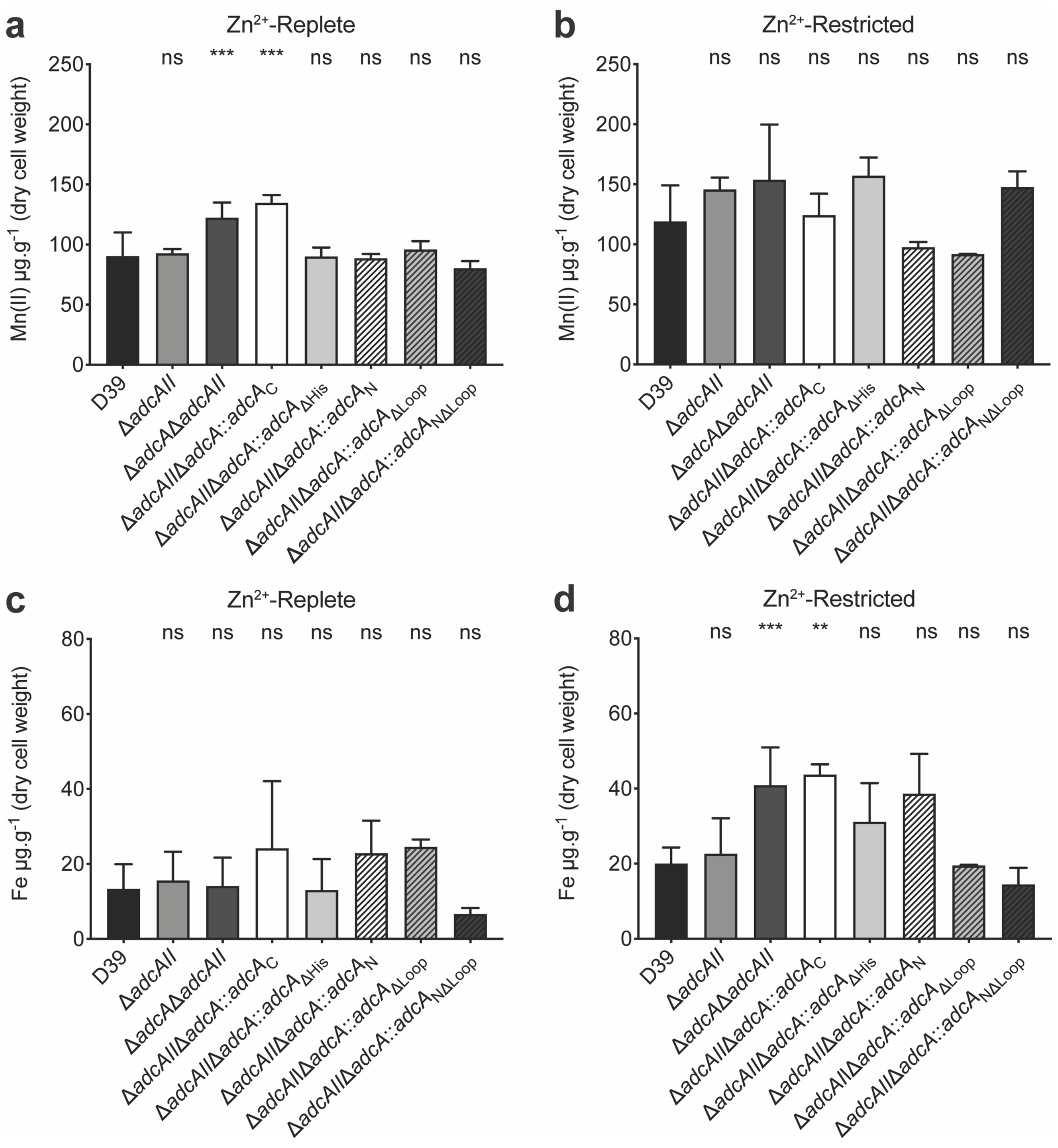

Supplementary Figure 4: Cellular metal accumulation of *S. pneumoniae* and mutant variants. Intracellular Zn(II) accumulation of *S. pneumoniae* D39 and the *adc* mutant strains in Zn^{2+}-replete (**a, c**) or Zn^{2+}-restricted (**b, d**) CDM as determined by ICP-MS. Data correspond to mean (± S.E.M.) µg metal/g dry-cell weight from duplicate measurements of at least 3 independent biological experiments. Statistical significance was determined by a one-way ANOVA with Tukey post-test (ns corresponds to P value > 0.05).
