# Supplementary material for: A Trap-Door Mechanism for Zinc Acquisition by Streptococcus pneumoniae AdcA
Source: mBio. 2021 Feb 2;12(1):e01958-20. doi: 10.1128/mBio.01958-20 (PMC7858048; doi:10.1128/mBio.01958-20)
Supplement: FIG S5 [file mBio.01958-20-sf005.pdf]

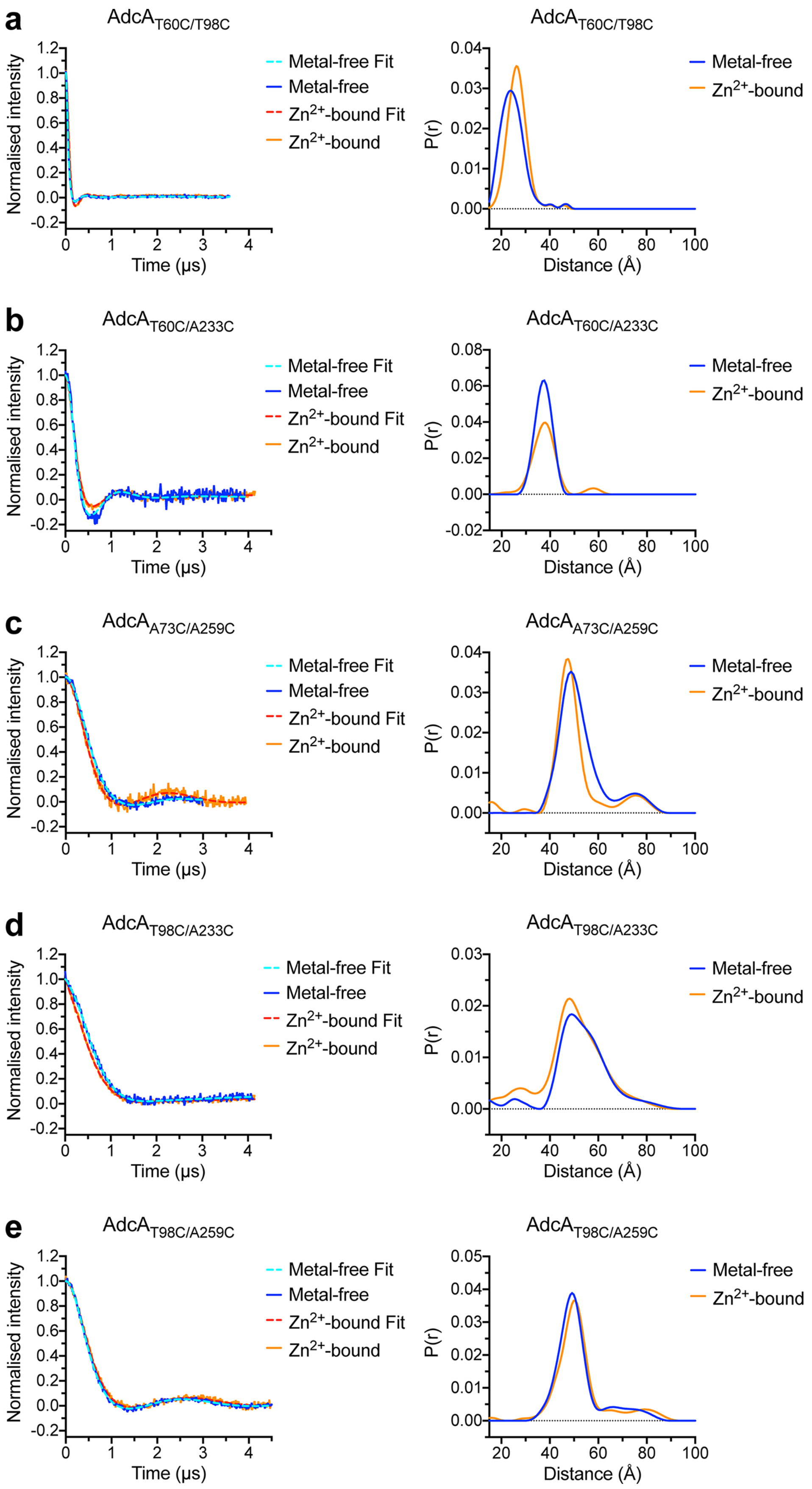

Supplementary Figure 5: Q-band DEER traces and distance distributions. Experimental Q-band (34 GHz) four-pulse DEER traces and the corresponding distance distributions for the cysteine-

containing variants of metal-free and Zn^{2+}-bound AdcA. Left panels show the DEER trace and the fit, obtained by Tikhonov regularisation, while the right panels display the corresponding distance distribution. The data are for $AdcA_{T60C/T98C}$ (**a**), $AdcA_{T60C/A233C}$ (**b**), $AdcA_{A73C/A259C}$ (**c**), $AdcA_{T98C/A233C}$ (**d**) and $AdcA_{T98C/A259C}$ (**e**).
