# Supplementary material for: A Trap-Door Mechanism for Zinc Acquisition by Streptococcus pneumoniae AdcA
Source: mBio. 2021 Feb 2;12(1):e01958-20. doi: 10.1128/mBio.01958-20 (PMC7858048; doi:10.1128/mBio.01958-20)
Supplement: FIG S6 [file mBio.01958-20-sf006.pdf]

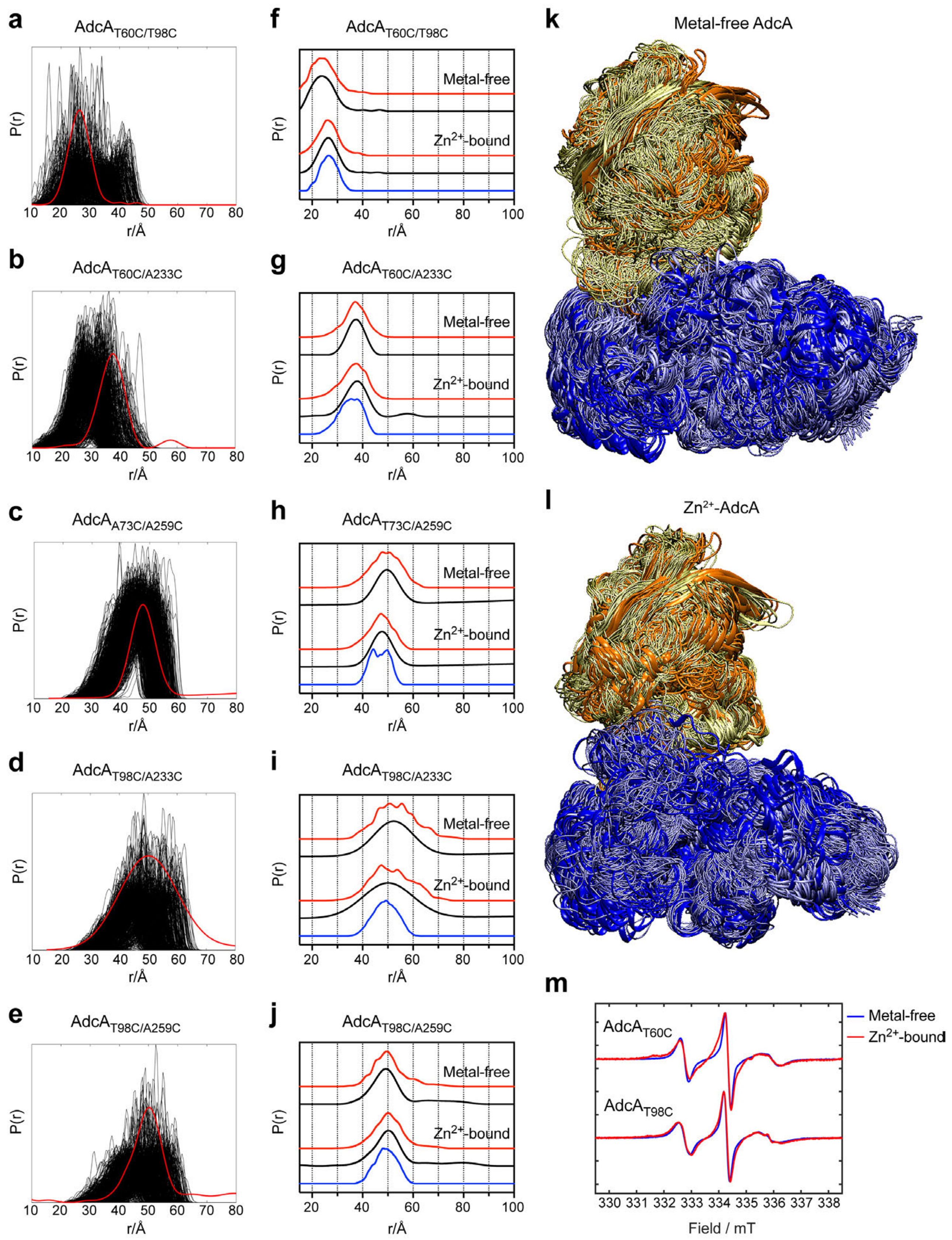

Supplementary Figure 6: DEER and MD structure distance distribution comparisons. DEER distance distributions for Zn^{2+}-bound $AdcA_{T60C/T98C}$ (**a**), $AdcA_{T60C/A233C}$ (**b**), $AdcA_{A73C/A259C}$ (**c**), $AdcA_{T98C/A233C}$ (**d**) and $AdcA_{T98C/A259C}$ (**e**) measured in frozen-solution (red line), overlaid with

distance distributions generated from room temperature MD simulations. Shown are the MD distance distributions generated from one 750 ns simulation (i.e. 750 structures, pdb files). Each of the MD structures was spin-labelled using MMM and distance distributions calculated. Distance distributions between the NO•-NO• of MTSSL incorporated into cysteine-containing variants of metal-free and Zn^{2+}-bound $AdcA_{T60C/T98C}$ (**f**), $AdcA_{T60C/A233C}$ (**g**), $AdcA_{A73C/A259C}$ (**h**), $AdcA_{T98C/A233C}$ (**i**) and $AdcA_{T98C/A259C}$ (**j**). Black line: experimental distance distributions computed from its DEER trace (panels a-e). Red line: fit to the DEER data computed from the set of distance distributions generated from the five molecular dynamics runs (5 runs, 750 conformations in each run, MTSSL added using MMM after the MD computations). Blue line: Distance distribution computed using the Zn^{2+}-bound AdcA crystal structure and adding MTSSL *in silico* using MMM. Cartoon representation of the set of MD structures that give the best global fit to the set of DEER distance distributions for metal-free AdcA (**k**) and Zn^{2+}-AdcA (**l**). (**m**) X-band (9.377 GHz) CW-EPR spectra measured in solution for metal-free (blue) and Zn^{2+}-bound (red) $AdcA_{T60C}$ and $AdcA_{T98C}$. The broader EPR line indicate reduced spin label mobility.
