# Supplementary material for: A Trap-Door Mechanism for Zinc Acquisition by Streptococcus pneumoniae AdcA
Source: mBio. 2021 Feb 2;12(1):e01958-20. doi: 10.1128/mBio.01958-20 (PMC7858048; doi:10.1128/mBio.01958-20)
Supplement: TABLE S3 [file mBio.01958-20-st003.pdf]

Supplementary Table 3A. Oligonucleotide primers used in this study

Name	Sequence (5' → 3')
adcA_LIC1F	TGGGTGGTGGATTTCCTGGTAAACTCAATATCGTGACA
adcA_LIC1R	TTGGAAGTATAAATTTCCATGCGCCAACATTTCTTGGGC
adcA_LIC2F	TGGGTGGTGGATTTCCTCAAAATGGTTACTTCGAGGAT
adcA_LIC2R	TTGGAAGTATAAATTTCCGTCTGTTGTTTGTTTCAAAGC
adcA_H63A_1F	GGTGCTGGGACAGAACCTGCGGAATACGAACCATCTGCC
adcA_H63A_1R	GGCAGATGGTTCGTATTCCGCAGGTTCTGTCCCAGCACC
adcA_H204A_1F	AACAAAAGAGCTTTGTGACTCAAGCGGCAGCCTTTAACTATCTTGCC
adcA_H204A_1R	GGCAAGATAGTTAAAGGCTGCCGCTTGAGTCACAAAGCTCTTTTGTT
adcA_A73C_1F	ACCATCTGCCAAGGCAGTTTGCAAAATCCAAGATGCAGAT
adcA_A73C_1R	ATCTGCATCTTGGATTTTGCAAACTGCCTTGGCAGATGGT
adcA_T98C_1F	AACATGGGTACCTAAATTGCTAGATTGTTTGGATAAGAAAAAAGTGAAAACC
adcA_T98C_1R	GGTTTTCACTTTTTTCTTATCCAAACAATCTAGCAATTTAGGTACCCATGTT
adcA_A233C_1F	CAGATGCAGAGCCATCAGCTTGTCGCTTGGCAG
adcA_A233C_1R	CTGCCAAGCGACAAGCTGATGGCTCTGCATCTG
adcA_A259C_1F	TCTATTTTGAAGAAAATGCCTCACAATGCCTTGCTAACACACTTTCAAAAG
adcA_A259C_1R	CTTTTGAAAGTGTGTTAGCAAGGCATTGTGAGGCATTTTCTTCAAAATAGA
adcA_T60C_1F	AACTCCTAATCGGTGCTGGGTGCGAACCTCATGAATACGAACC
adcA_T60C_1R	GGTTCGTATTCATGAGGTTCGCACCCAGCACCGATTAGGAGTT
adcA_F35C_1F	AAACTCAATATCGTGACAACCTGTTACCCTGTCTATGAATTTACC
adcA_F35C_1R	GGTAAATTCATAGACAGGGTAACAGGTTGTCACGATATTGAGTTT
adcA_A58C_1F	GGCTAATGTAGAACTCCTAATCGGTTGTGGGACAGAACCT
adcA_A58C_1R	AGGTTCTGTCCCACAACCGATTAGGAGTTCTACATTAGCC
adcA_XC	GCAGATGGTCAAAATGGTTACTTCGAGGATGCAG
adcA_CF	ACCATTTTGACCATCTGCCTGTTTTTGATTGG
rpsL_F	GGTCGGAATTGTAGCTAACAG
rpsL_R	CTACACGTCCACGACGAGCAG
Janus_F	CCGTTTGATTTTTAATGGATAATG
Janus_R	AGAGACCTGGGCCCCTTTCC
adcA_seq_F	CTAGTATTGCCCTGCGTCTG
adcA_seq_R	AGCCCTTAACTATGGCACTAG
adcA_Flank_F	TCCTAATAGGTCATTGCACTAGC
adcA_Flank_R	TTGTGCTATAATATAGTTGAAATGATAAAT
adcA_Janus_X	CATTATCCATTAAAAATCAAACGGGTTCGTCTCCTATTTGATAAAACG
adcA_Janus_Y	AGGGGCCCAGGTCTCTTGAGAAATCGACTAGTTCATAAGAG
adcA_F	GGTAAACTCAATATCGTGACAAC
adcA_R1	ATGCGCCAACATTTCTT
adcA_R2	ATGAACTAGTCGATTTCTCATCAGTCTGTTGTTTGTTTCAAAGC
adcA_X	GTTGTCACGATATTGAGTTTACC
adcA_Y1	AATGTTGGCGCATTGA
adcA_Y2	TGATGAGAAATCGACTAGTTCAT

Supplementary Table 3B. Strains used in this study

**Bacterial Strains**	**Genotype**	**Source/Ref**
Streptococcus pneumoniae D39	Capsular serotype 2	NCTC7466
D39 Δ*adcA*	Replacement of *adcA* with cml^{R}	(1)
D39 Δ*adcAII*	Null mutant of *adcAII*	(1)
D39 Δ*adcA*Δ*adcAII*	Replacement of *adcA* with cml^{R}; null mutant of *adcAII*	(1)
D39 *rpsL*$^{+}$	Replacement of *rpsL* gene with K56T mutant variant	This study
D39 Δ*adcAII*Δ*adcA*::*Janus*	Replacement of *adcA* with the Janus cassette; null mutant of Δ*adcAII*	This study
D39 Δ*adcAII*Δ*adcA*::*adcA*$_{\mathrm{N}}$	*adcA* truncated to encode only AdcA$_{\mathrm{N}}$ domain (residues 1 to 308); null mutant of Δ*adcAII*	This study
D39 Δ*adcAII*Δ*adcA*::*adcA*$_{\mathrm{C}}$	*adcA* truncated to encode only AdcA$_{\mathrm{C}}$ domain (residues 1 to 27 and 326 to 501); null mutant of Δ*adcAII*	This study
D39 Δ*adcAII*Δ*adcA*::*adcA*$_{\Delta\mathrm{Loop}}$	*adcA* mutated to exclude His-rich loop (residues 120 to 136 removed except Gly124, Gly129 and Gly132); null mutant of Δ*adcAII*	This study
D39 Δ*adcAII*Δ*adcA*::*adcA*$_{\mathrm{N}\Delta\mathrm{Loop}}$	*adcA* truncated to encode only AdcA$_{\mathrm{N}}$ domain (residues 1 to 308); His-rich loop excluded (as above); null mutant of Δ*adcAII*	This study
D39 Δ*adcAII*Δ*adcA*::*adcA*$_{\Delta\mathrm{His}}$	*adcA* mutated in the metal-coordinating pocket of the AdcA$_{\mathrm{N}}$ domain (H63A, H140A, H204A); null mutant of Δ*adcAII*	This study
D39 Δ*adcAII*Δ*adcA*::*adcA*$_{\mathrm{H63A}}$	*adcA* H63A variant; null mutant of Δ*adcAII*	This study
D39 Δ*adcAII*Δ*adcA*::*adcA*$_{\mathrm{H204A}}$	*adcA* H204A variant; null mutant of Δ*adcAII*	This study

Supplementary Table 3C. Plasmids used in this study.

Name	Description	Source
pCAM-nLIC01	Kan^{R}, ligation independent cloning expression vector encoding a N-terminal dodecahistidine tag	(1)
pCAM-cLIC01	Kan^{R}, ligation independent cloning expression vector encoding a C-terminal dodecahistidine tag	(1)
pCAM-nLIC01-AdcA	Kan^{R}, *adcA* gene lacking the signal sequence (residues 1 to 26), cloned into pCAM-nLIC01 vector	(1)
pCAM-cLIC01-AdcA$_{C}$	Kan^{R}, *adcA* gene lacking the signal sequence and the AdcA$_{N}$ domain, cloned into pCAM-cLIC01 vector	This study
pCAM-nLIC01-AdcA$_{H63A}$	Kan^{R}, *adcA* gene lacking the signal sequence with a H63A mutation, cloned into the pCAM-nLIC01 vector	This study
pUC57-AdcA$_{\Delta Loop}$	Kan^{R}, *adcA* gene lacking the signal sequence and His-rich loop (residues 120 to 136 except Gly124, Gly129 and Gly132), in the pUC57 vector.	This study
pCAM-nLIC01-AdcA$_{\Delta Loop}$	Kan^{R}, synthesised *adcA* gene from pUC57 vector lacking the signal sequence and the His-rich loop (as above), cloned into pCAM-nLIC01 vector	This study
pCAM-nLIC01-AdcA$_{N}$	Kan^{R}, *adcA* gene lacking the signal sequence, the AdcA$_{C}$ domain, cloned into pCAM-nLIC01 vector	This study
pCAM-nLIC01-AdcA$_{N\Delta Loop}$	Kan^{R}, synthesised *adcA* gene lacking the signal sequence, the AdcA$_{C}$ domain and the His-rich loop (as above), cloned into pCAM-nLIC01 vector	This study
pCAM-nLIC01-AdcA$_{N\ H63A}$	Kan^{R}, *adcA* gene lacking the signal sequence and the AdcA$_{C}$ domain, with His63A mutation, cloned into pCAM-nLIC01 vector	This study
pCAM-nLIC01-AdcA$_{N\ H204A}$	Kan^{R}, *adcA* gene lacking the signal sequence and the AdcA$_{C}$ domain, with His204A mutation, cloned into pCAM-nLIC01 vector	This study
pCAM-nLIC01-AdcA$_{\Delta His}$	Kan^{R}, *adcA* gene lacking the signal sequence, with His63A, His140A and His204A mutations, cloned into pCAM-nLIC01 vector	This study
pCAM-nLIC01-AdcA$_{T60C}$	Kan^{R}, *adcA* gene lacking the signal sequence, with a T60C mutation, cloned into pCAM-nLIC01 vector	This study
pCAM-nLIC01-AdcA$_{T98C}$	Kan^{R}, *adcA* gene lacking the signal sequence, with a T98C mutation, cloned into pCAM-nLIC01 vector	This study
pCAM-nLIC01-AdcA$_{T98C/A233C}$	Kan^{R}, *adcA* gene lacking the signal sequence, with T98C and A233C mutations, cloned into pCAM-nLIC01 vector	This study

pCAM-nLIC01-AdcA$_{A73C/A259C}$	Kan^{R}, *adcA* gene lacking the signal sequence, with A73C and A259C mutations, cloned into pCAM-nLIC01 vector	This study
pCAM-nLIC01-AdcA$_{T60C/T98C}$	Kan^{R}, *adcA* gene lacking the signal sequence, with T60C and T98C mutations, cloned into pCAM-nLIC01 vector	This study
pCAM-nLIC01-AdcA$_{T60C/A233C}$	Kan^{R}, *adcA* gene lacking the signal sequence, with T60C and A233C mutations, cloned into pCAM-nLIC01 vector	This study
pCAM-nLIC01-AdcA$_{T98C/A259C}$	Kan^{R}, *adcA* gene lacking the signal sequence, with T98C and A259C mutations, cloned into pCAM-nLIC01 vector	This study
